# Stereoselective Synthesis, Pro-resolution, and Anti-inflammatory Actions of RvD5_n-3 DPA_

**DOI:** 10.1021/acs.jnatprod.3c00769

**Published:** 2023-10-25

**Authors:** Karina Ervik, Amalie F. Reinertsen, Duco S. Koenis, Jesmond Dalli, Trond V. Hansen

**Affiliations:** † Department of Pharmacy, Section for Pharmaceutical Chemistry, University of Oslo, P.O. Box 1068, 0316 Oslo, Norway; ‡ Lipid Mediator Unit, Center for Biochemical Pharmacology, William Harvey Research, Institute, Barts and The London School of Medicine, Queen Mary University of London Charterhouse Square, London EC1M 6BQ, U.K.

## Abstract

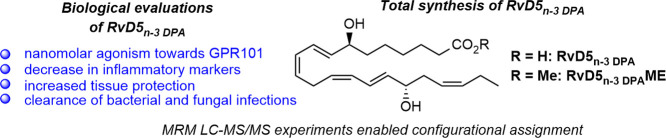

The methyl ester of resolvin D5_n-3 DPA_, a lipid mediator biosynthesized from the omega-3 fatty acid n-3 docosapentaenoic acid, was stereoselectively prepared in 8% yield over 12 steps (longest linear sequence). The key steps for the introduction of the two stereogenic secondary alcohols were an organocatalyzed oxyamination and the Midland Alpine borane reduction. For the assembly of the carbon chain, the Sonogashira cross-coupling reaction and the Takai olefination were utilized. The physical properties, including retention time in liquid chromatography and tandem mass spectra, of the synthetic material were matched against material from human peripheral blood and mouse infectious exudates. Synthetic RvD5_n-3 DPA_, obtained just prior to biological experiments, displayed potent leukocyte-directed activities, upregulating the ability of neutrophils and macrophages to phagocytose bacteria, known as hallmark bioactions of specialized pro-resolving endogenous mediators.

The acute inflammatory response is essential for protection against injury or pathogenic infections.^1^ This self-limited and localized process is divided into an initiation and a resolution phase.^2^ The resolution phase is essential to curtail inflammation and restore tissue homeostasis by elimination of harmful agents and cell debris.^3^ Normally, the acute inflammatory response is self-limited and resolves without interruption. However, if unresolved, a chronic inflammatory situation may result and develop further into human diseases, such as rheumatoid arthritis, ulcerative colitis, cardiovascular disorders, and Parkinson’s and Alzheimer’s diseases.^4^ It is now evident that actively induced biochemical pathways counter-regulate inflammation and promote resolution at the site of infection or injury.^5^ Hence, resolution is an active rather than a passive process, as once believed, where oxygenated polyunsaturated lipid mediators are essential.^6^ These mediators, named specialized pro-resolving mediators (SPMs), have been isolated from diverse natural sources. SPMs are actively biosynthesized upon demand from the ω-6 polyunsaturated fatty acid (PUFA) arachidonic acid and the ω-3 PUFAs eicosapentaenoic acid (EPA), docosahexaenoic acid (DHA), and n-3 docosapentaenoic acid (n-3 DPA).^7−9^ The ability of SPMs to govern inflammatory resolution processes is considered a biomedical paradigm shift.^10^ SPMs are nontoxic and potent agonists that stereoselectively activate G-protein coupled receptors (GPCRs).^11,12^ Examples of SPMs are the DHA-derived resolvins, protectins and maresins.^13^ More recently SPMs biosynthesized from n-3 DPA have emerged with interesting biological properties.^7,14−16^ Some examples are shown in Figure 1.

**Figure 1:**
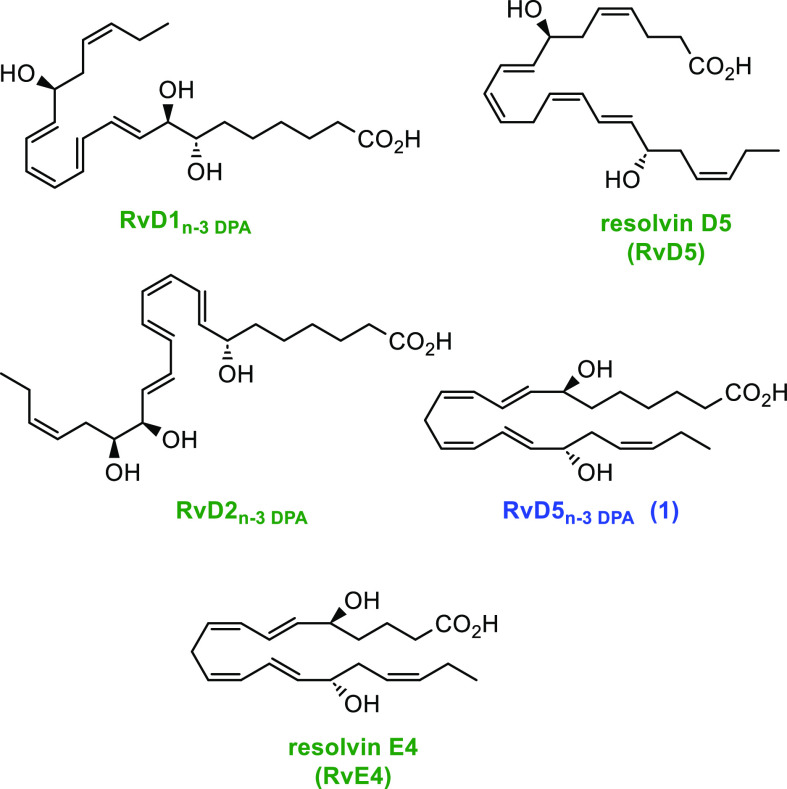
Chemical structures of some resolvins derived from n-3 DPA, and the DHA-derived RvD5 and RvE4 biosynthesized from EPA.

Because SPMs enable resolution of inflammation without immunosuppression or toxic effects, these naturally occurring compounds have gained great interest in drug discovery.^10,11^ However, such efforts depend on the stereoselective total synthesis of SPMs for further biological studies. Moreover, for configurational assignments, matching experiments using LC-MS/MS are in demand, as SPMs are formed in nano- to picogram amounts, too low for NMR configurational studies.^5,16,17^

As of today, stereoselective total syntheses of RvD1_n-3 DPA_^18^ and RvD2_n-3 DPA_^19^ have been achieved (Figure 1). Recently, it was reported that RvD5_n-3 DPA_ (**1**) binds to and activates GPR101, exerting tissue-protective actions during inflammatory arthritis.^15^ This SPM is formed after two consecutive lipoxygenation reactions, followed by hydroperoxide reduction by a peroxidase (Scheme 1). In the detailed anticipated biogenetic formation of **1**, the first biosynthetic step in the presence of 15-lipoxygenase (LOX) forms 17(*S*)-H*p*DPA, while the second lipoxygenation step is catalyzed by 5-LOX. This product, 7(*S*),17(*S*)-diH*p*DPA, is then directly reduced to give SPM **1**. Alternatively, an epoxide intermediate could be formed that is enzymatically hydrolyzed to either RvD1_n-3 DPA_ or RvD2_n-3 DPA_ (Scheme 1).

**Scheme 1:**
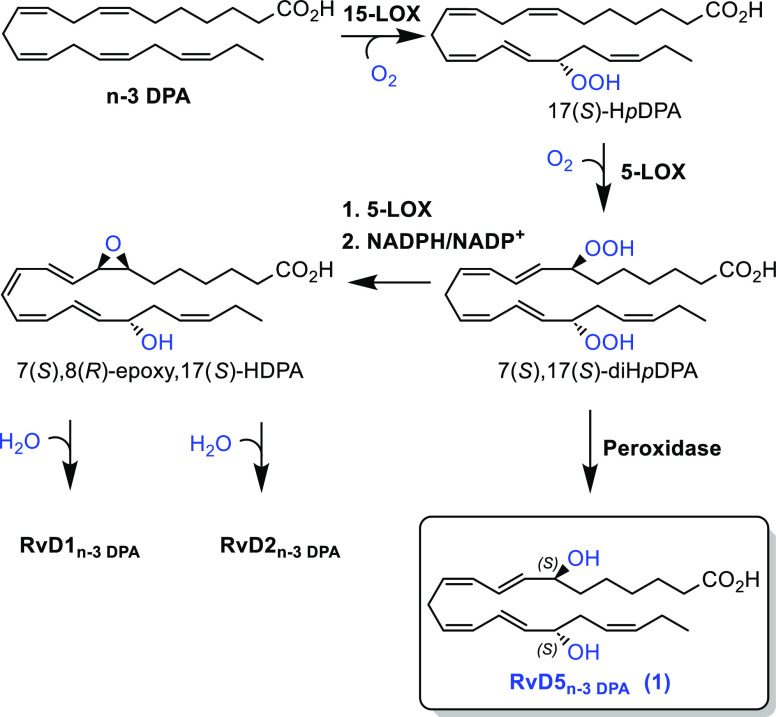
Proposed Biosynthesis of RvD5_n-3 DPA_ (**1**), RvD1_n-3 DPA_, and RvD2_n-3 DPA_ from n-3 DPA

The DHA-derived SPM RvD5 (Figure 1) was isolated from inflammatory exudates, and its structure was established by UV data, LC-MS/MS experiments,^8^ and total synthesis.^20,21^ RvD5 was first detected in leukocytes, brain cells, and glia cells,^8^ and later it was also detected in several patient models.^22^ RvD5 given to mice infected with *Escherichia coli* has shown a significantly enhanced phagocyte containment of *E. coli* compared to mice infected with *E. coli* alone (160% increase).^23^ In addition, it showed protection from hypothermia and overall increased survival in *E. coli*-infected mice. RvD5 also reduced pro-inflammatory cytokines such as KC and TNF-α.^23^ Further studies revealed that RvD5 plays a role in downregulating a panel of inflammation-related genes, including NF-κB, phosphodiesterase 4B (PDE4B), and COX-2, which might contribute to their actions in enhancing phagocytosis and bacterial clearance in vivo.^23^ The biologically interesting effects reported for the DHA-derived SPMs spurred an interest in investigating the biosynthetic formation of n-3 DPA-derived SPMs.^7^ The congener RvD5, RvD5_n-3 DPA_ (**1**), was first reported in 2013. LC-MS/MS data, physical properties (MS and UV–vis data), and biosynthetic considerations enabled the tentative structure elucidation of **1** as depicted in Scheme 1.^7^ This SPM contains two highly sensitive *E*,*Z*-dienes, both adjacent to stereogenic allylic alcohols that are prone to elimination reactions (Scheme 2). Based on our successful synthesis of RvE4,^24^ the retrosynthetic proposal outlined in Scheme 2 seemed attractive to attempt. This analysis identified three key fragments, **3**, **4**, and **5**, as suitable building blocks, wherein the linchpin **4** is commercially available. The chemically labile *Z*,*Z*-skipped diene present in **1** and **2** should be introduced late in any total synthesis efforts.

**Scheme 2:**
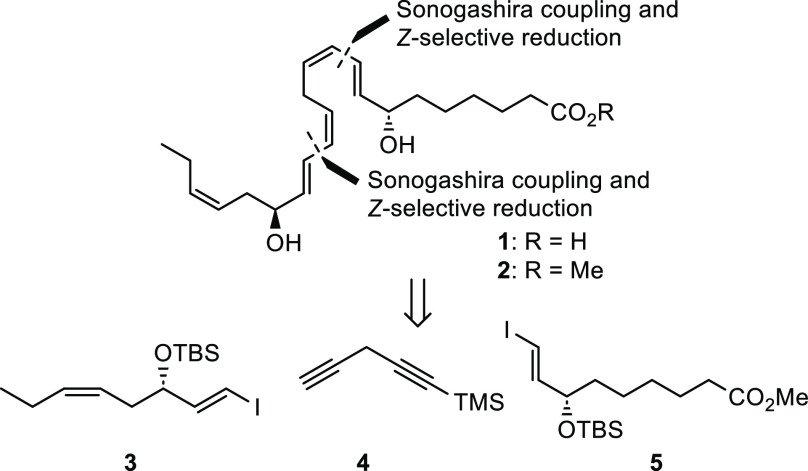
Overview of the Retrosynthetic Analysis of RvD5_n-3 DPA_ (**1**) and Its Methyl Ester **2**

## Results and Discussion

**Scheme 3:**
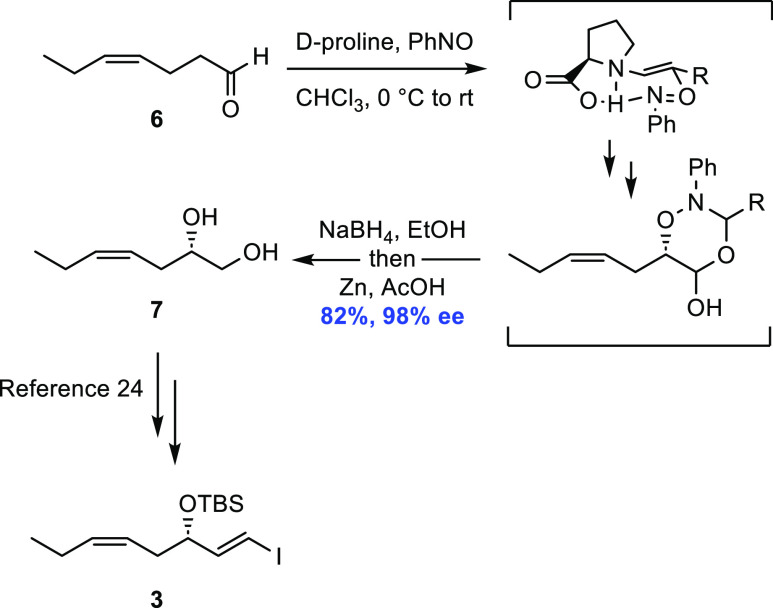
Preparation of Vinyl Iodide **3**

Despite the apparent similar structural features between RvE4 and RvD5_n-3 DPA_ (**1**), we experienced that SPM **1** is chemically more sensitive than RvE4. The same observations were also applied for some of the intermediates. However, the total synthesis was initiated with the preparation of the ω-3 fragment **3** from affordable *cis*-4-heptenal (**6**) (Scheme 3), utilizing a protocol developed by the MacMillan group^25^ and essentially as earlier reported in our total synthesis of RvE4.^24^ This enantioselective, organocatalytic α-oxyamination reaction afforded diol **7** in 82% yield and 98% enantiomeric excess (*ee*). This diol was converted, via a highly *E*-selective Takai olefination in the last step, to vinyl iodide **3** (Scheme 3). Diastereomerically pure **3** was obtained after chromatographic purification.

The synthesis of vinyl iodide **5** was recently presented^19^ and was resynthesized in order to investigate the outlined Sonogashira cross-coupling reactions.^26^ This reaction has also been successfully applied in total synthesis of SPMs,^27^ including RvD5.^20^

**Scheme 4:**
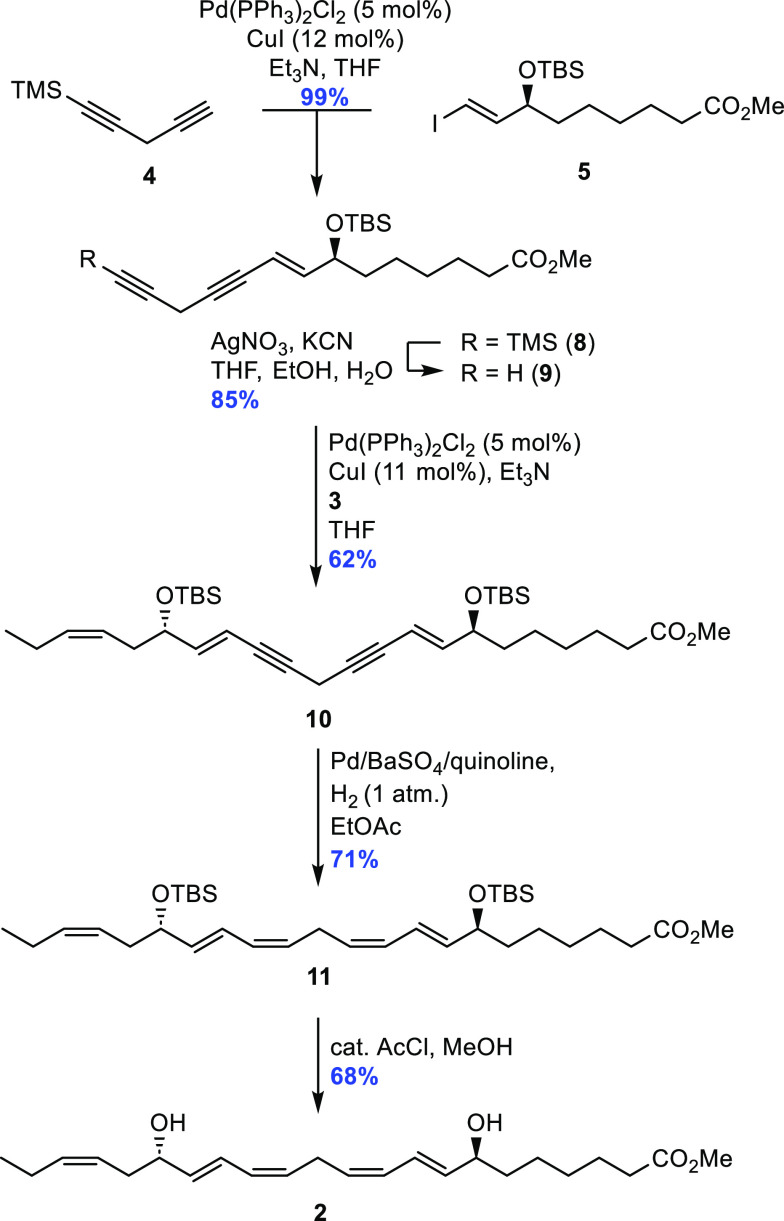
Sonogashira Cross-Coupling Reactions and *Z*-Selective Hydrogenation to Complete the Synthesis of RvD5_n-3 DPA_ Methyl Ester (**2**)

The first reaction was performed with the linchpin **4** and catalytic amounts of Pd(PPh_3_)_2_Cl_2_ (5 mol %) and CuI (12 mol %), which provided a high yield of the coupled product **8** (Scheme 4). Due to the inherent lability of the diyne system in **8**, TMS deprotection was accomplished utilizing the mild reaction conditions of AgNO_3_ and KCN,^28^ yielding the terminal alkyne **9** in 85% yield. The same Sonogashira cross-coupling conditions were then tried for the final carbon–carbon bond-forming reaction between alkyne **9** and vinyl iodide **3**. Unfortunately, these reaction conditions afforded product **10** in a disappointingly low yield of 34%. Therefore, the classic Sonogashira coupling conditions, with Pd(PPh_3_)_4_ (3 mol %) and CuI (6 mol %), were attempted, but the coupled product **10** was not observed. However, vinyl iodide **3** was reisolated in 90% yield, while terminal alkyne **9** no longer could be detected in the reaction mixture. This indicated decomposition of the alkyne or undesired side reactions such as homocoupling of the alkyne and further decomposition. Nevertheless, no homocoupled byproduct was isolated, strengthening the notion of rapid decomposition of diyne **9**. Such challenges were not observed in the preparation of RvE4.^24^ With this information, the first Sonogashira reaction conditions containing Pd(PPh_3_)_2_Cl_2_ (5 mol %) and CuI (11 mol %) was tried once more, with the equivalent of diyne **9** increased from 1.2 to 2.5. These conditions gave the desired coupled product **10** in an isolated 62% yield.

Then our tried-and-tested *Z*-selective Lindlar hydrogenation protocol was first attempted for the diyne system in **10**,^25,29^ but HRMS analyses of the reaction mixture revealed no reduction of the triple bonds. This was a surprise, as Rodriguez and Spur successfully used a Lindlar reduction in their total synthesis of RvD5,^20^ although on a slightly different diyne than **10**. A search in the literature gave interest in the Pd/BaSO_4_/quinoline hydrogenation protocol.^30,31^ These conditions were applied and showed rapid and selective reduction of the two triple bonds. Slight over-reduction was observed; however, isolation of product **11** in 71% yield was achieved after column chromatography. Removal of the two TBS groups was successfully accomplished by subjecting **11** to a catalytic amount of acetyl chloride in MeOH,^32^ yielding the RvD5_n-3 DPA_ methyl ester **2** in 68% yield. These mild conditions were used instead of the more well-known tetra-*n*-butylammonium fluoride (TBAF) in THF due to reported byproduct formation and difficulties experienced during the purification process of RvE4.^24^ The ester **2** was obtained in 68% yield (97%, HPLC analysis) after chromatographic purification with NMR (^1^H, ^13^C, and COSY), HRMS, and UV data in accordance with the structure of **2** (Supporting Information). Analyses of the ^1^H NMR data (*J* = 15.7 Hz for H8/H16, *J* = 15.0 Hz for H9/H15, and *J* = 10.8 Hz for H10/H11) and the COSY spectrum confirmed the olefin configurations of the two *E*,*Z*-dienes in **2** (Figure 1), which were further supported by the UV data (λ_max_ = 242 nm; log ε = 4.64).^20^

**Figure 2:**
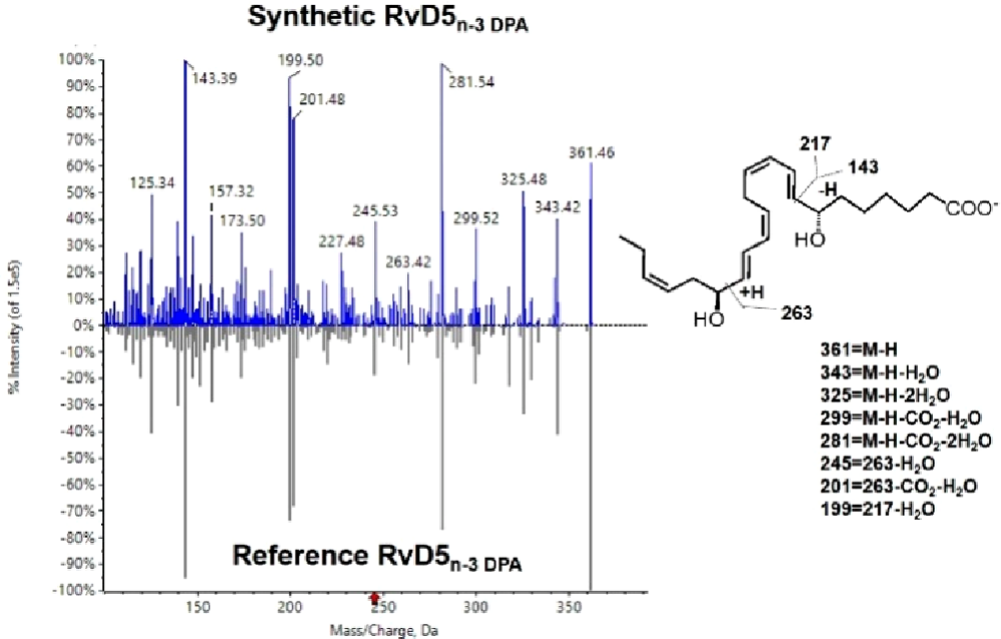
MS/MS fragmentation spectrum of the synthetic material of **1** matched that of the reference spectrum for biologically produced RvD5_n-3 DPA_ (**1**). The tandem mass spectrum of synthetic **1** was compared with that of reference material using a Sciex QTRap 6500+ and Sciex OS Library Match function.

We next evaluated whether the physical properties of the synthetic material matched those of biological RvD5_n-3 DPA_. Using the free acid of the synthetic material **2**, obtained just prior to analyses as described in the Experimental Section, due to the chemical sensitivity of SPMs,^5,33^ we first evaluated whether the MS/MS spectrum matched that of the reference material. Here we observed that the MS/MS spectrum of synthetic **1** matched that of the reference material including key fragments such as *m*/*z* 143, 199, and 263 (Figure 2). The UV data for synthetic **1** (λ_max_ = 245 nm; log ε = 4.64, EtOH) were in accordance with the literature for biogenetic **1**(7a,^15^) and its congener RvD5 (λ_max_ = 245 nm).^8,20^ Similar data have been reported for RvE4, which contains the same olefin configuration.^24^ Moreover, UV data for *E*,*E*-configured oxygenated lipids show hypochromic effects.^34^

**Figure 3:**
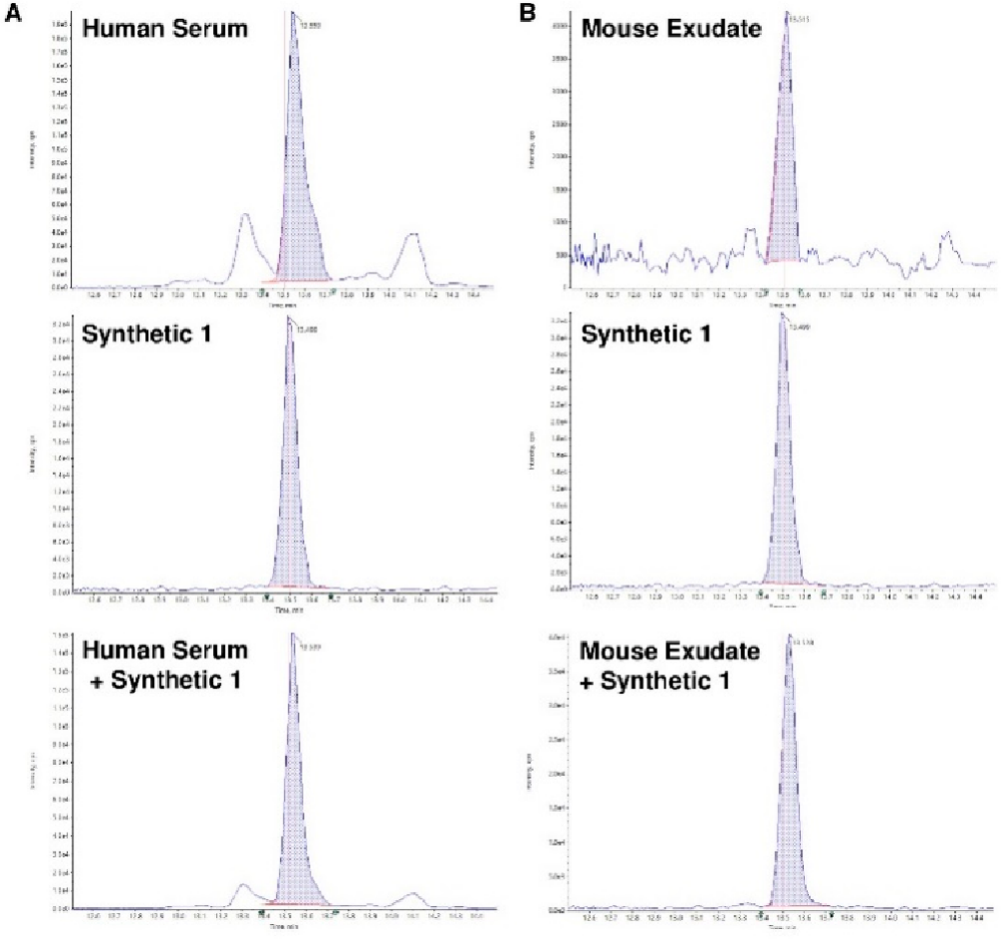
Chromatographic properties of synthetic **1** matched those of biogenetic RvD5_n-3 DPA_. Multiple reaction chromatograms for selected ion pairs *m*/*z* 361 > 199 for (A) human serum and (B) mouse exudates. Top panels report the traces for the biological sample, while center panels report the chromatographic trace for the synthetic **1**, and bottom panels report the co-injection of the synthetic with the biological materials. Peaks highlighted in blue shading correspond to the RvD5_n-3 DPA_. Results are representative of *n* = 3 determinations for human serum samples and *n* = 3 mice for exudate samples.

Because we recently reported that RvD5_n-3 DPA_ is produced in human peripheral blood,^15^ we next sought to compare the chromatographic properties of the synthetic material of **1** with those of endogenous RvD5_n-3 DPA_ from human serum. Using synthetic **1** we observed that in RP-LC-MS/MS experiments both the biological and synthetic RvD5_n-3 DPA_ (**1**) gave a peak in the multiple reaction monitoring (MRM) transition 361 > 199 with a retention time (*t*_r_) of 13.5 min (Figure 3A). Furthermore, spiking of the biological material with **1** gave a single peak that eluted with a *t*_r_ of 13.5 min (Figure 3A). Similar results were obtained with mouse infectious exudates where we observed a peak in the 361 > 199 MRM transition with a *t*_r_ of 13.5 min. Furthermore, spiking of the synthetic material of **1** into the biological material yielded a single peak in the 361 > 199 MRM transition with a *t*_r_ of 13.5 min (Figure 3B).

**Figure 4:**
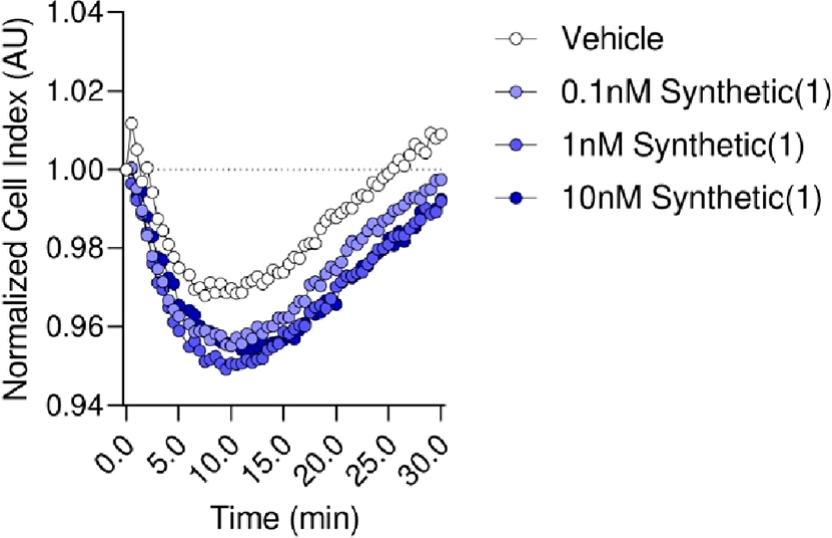
Synthetic **1** activates human GPR101. GPR101-expressing CHO cells were incubated with the indicated concentrations with synthetic **1** or vehicle (DMEM/F-12 medium with 0.05% EtOH), and impedance was measured for 30 min using the xCelligence RTCA DP system. Results are representative of three independent experiments, with four replicates per experiment.

Having observed that the physical properties of synthetic material **1** matched those of the endogenous and reference material, we next sought to determine whether synthetic material **1** also carried the biological properties of RvD5_n-3 DPA_. We recently found that RvD5_n-3 DPA_ activates the orphan receptor GPR101 to exert its biological activities.^15^ Therefore, we assessed whether synthetic **1** displayed agonistic activities with respect to this receptor. Using an impedance-based assay, we observed that synthetic **1** potently activated GPR101 in the 0.1 to 10 nM range, as denoted by a change in impedance in cells overexpressing the receptor (Figure 4).

**Figure 5:**
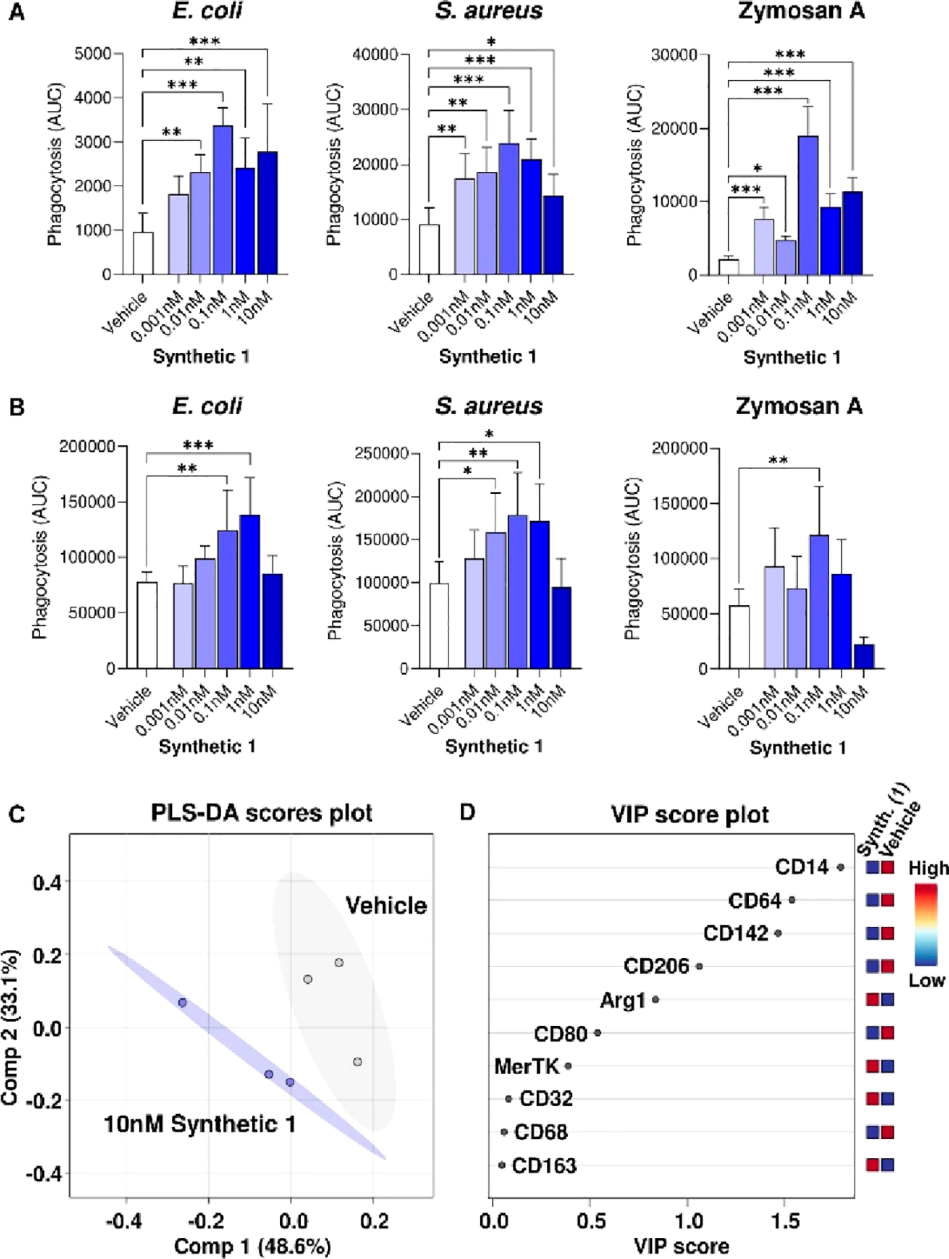
Synthetic **1** regulates neutrophil and macrophage responses. Human (A) neutrophils and (B) monocyte-derived macrophages were incubated with the indicated concentrations of synthetic **1** for 15 min at 37 °C in serum-free RPMI-1640 medium, followed by addition of fluorescently labeled *E. coli*, *S. aureus*, or zymosan A particles, and phagocytosis was evaluated using high-content imaging. Results are mean ± SEM, *N* = 6 healthy volunteers. (C, D) Human monocytes were incubated with synthetic **1** (10 nM) and differentiated to macrophages by incubating with GM-CSF (20 ng/mL) in RPMI-1640 medium containing 10% human serum for 7 days. Expression of phenotypic markers was then evaluated using flow cytometry, and median fluorescence intensity values for macrophage activation markers were analyzed by (C) partial least-squares discriminant analysis (PLS-DA), followed by (D) variable importance in projection (VIP) score calculation using MetaboAnalyst.

We next evaluated whether synthetic **1** regulated human phagocyte responses by assessing the ability of this molecule to upregulate the clearance of bacterial and fungal particles by human neutrophils and macrophages. Here we observed that SPM **1** upregulates phagocytosis of fluorescently labeled *E. coli*, *Staphylococcus aureus*, and yeast cell wall-derived zymosan A particles in both cell types, displaying a characteristic bell-shaped dose–response (Figure 5A,B). Furthermore, we observed that synthetic **1** also regulates the expression of macrophage phenotypic markers (Figure 5C), as human macrophages differentiated in the presence of synthetic **1** showed lower expression of markers linked with an inflammatory phenotype, such as CD142 and CD80, while it upregulated expression of proteins linked with tissue protection, such as Arginase-1, MerTK, and CD163 (Figure 5D). Altogether, the results presented in Figures 4 and 5 reveal that RvD5_n-3 DPA_ (**1**) possesses potent pro-resolution and anti-inflammatory actions as well as agonism toward GPR101. The regulation of neutrophils and macrophages to phagocytose bacteria that SPM **1** shows is of great interest in drug discovery efforts based on resolution pharmacology.

## Conclusion

In summary, a stereoselective total synthesis of methyl ester **2** of the biologically interesting specialized pro-resolving mediator RvD5_n-3 DPA_ (**1**) is presented. These efforts gave **2** in an 8% overall yield over 12 steps (longest linear sequence). Data from LC-MS/MS matching experiments enabled the configurational assignment of natural product **1** as (7*S*,8*E*,10*Z*,13*Z*,15*E*,17*S*,19*Z*)-7,17-dihydroxydocosa-8,10,13,15,19-pentaenoic acid. Biological evaluations of RvD5_n-3 DPA_ (**1**) demonstrated a potent upregulation of bacterial clearance and fungal particles in neutrophils and macrophages. These activities, together with downregulation of inflammation markers, show that this natural product possesses both pro-resolution and anti-inflammatory properties. In addition, nanomolar agonism toward GPR101 was observed, rendering additional support that RvD5_n-3 DPA_ (**1**) is an interesting lead for drug discovery^10,11^ based on resolution pharmacology.^12^

## Experimental Section

### General Experimental Procedures

Unless otherwise stated, all commercially available reagents and solvents were used in the form in which they were supplied without any further purification. The stated yields are based on the isolated material. All sensitive reactions were performed under an argon atmosphere by using Schlenk techniques. Reaction flasks were covered with aluminum foil during sensitive reactions and storage to minimize exposure to light. Thin layer chromatography was performed on silica gel 60 F254 aluminum-backed plates fabricated by Merck. Flash column chromatography was performed on silica gel 60 (40–63 μm) fabricated by Merck. Optical rotations were measured using a 0.2 mL cell with a 0.1 dm path length on a PerkinElmer 341 polarimeter. The UV–vis spectrum was recorded by using an Agilent Technologies Cary 8485 UV–vis spectrophotometer using quartz cuvettes. NMR spectra were recorded on a Bruker AVII 400 or AVII 600 spectrometer at 400 MHz/600 MHz for ^1^H NMR and at 101 MHz/151 MHz for ^13^C NMR. Chemical shifts are reported relative to the central residual protium solvent resonance in ^1^H NMR (CDCl_3_ = δ 7.27, CD_3_OD = δ 3.31, and C_6_D_6_ = δ 7.16) and the central carbon solvent resonance in ^13^C NMR (CDCl_3_ = δ 77.16, CD_3_OD = δ 49.0, and C_6_D_6_ = δ 128.1). High-resolution mass spectra were recorded at 70 eV on a Micromass Prospec Q or Micromass QTOF 2W spectrometer by using ESI as the method of ionization. HPLC analyses were performed using an AD-H stationary phase (CHIRALPAK, 4.6 mm × 250 mm, particle size 5 μm, from Daicel Corporation) or a C18 stationary phase (Eclipse XDBC18, 4.6 mm × 250 mm, particle size 5 μm, from Agilent Technologies), applying the conditions stated.

#### Methyl (*S*,*E*)-7-((*tert*-butyldimethylsilyl)oxy)-14-(trimethylsilyl)tetradeca-8-en-10,13-diynoate (**8**)

Vinyl iodide **5** (149 mg, 0.35 mmol, 1.00 equiv) was dissolved in THF (2.0 mL). The solution was cooled to 0 °C before Pd(PPh_3_)_2_Cl_2_ (12.3 mg, 0.017 mmol, 5 mol %), CuI (8.0 mg, 0.04 mmol, 12 mol %), and Et_3_N (71 mg, 0.10 mL, 0.70 mmol, 2.00 equiv) were added. Trimethyl(penta-1,4-diyn-1-yl)silane (**4**, 119 mg, 0.15 mL, 0.88 mmol, 2.50 equiv) was dissolved in THF (0.50 mL) and added dropwise. The reaction mixture was allowed to slowly warm to ambient temperature and stirred in the dark for an additional 16 h. After completion, the reaction mixture was filtered through a short plug of silica gel (15% EtOAc in heptane) and concentrated in vacuo. The crude product thus obtained was purified by flash column chromatography (SiO_2_, 5% EtOAc in heptane) to obtain coupled product **8** (151 mg, 0.34 mmol, 99%) as a yellow oil: *R*_f_ (5% EtOAc in heptane, visualized by KMnO_4_ stain) = 0.31; [α]_D_^25^ −0.14 (*c* 0.4, CH_2_Cl_2_); ^1^H NMR (400 MHz, CDCl_3_) δ 6.05 (dd, *J* = 15.8, 5.5 Hz, 1H), 5.61–5.56 (m, 1H), 4.12–4.10 (m, 1H), 3.64 (s, 3H), 3.31 (d, *J* = 2.2 Hz, 2H), 2.27 (t, *J* = 7.5 Hz, 2H), 1.61–1.57 (m, 2H), 1.46–1.42 (m, 2H), 1.30–1.23 (m, 9H), 0.86 (s, 9H), 0.14 (s, 9H), 0.01 (s, 3H), 0.00 (s, 3H); ^13^C NMR (101 MHz, CDCl_3_) δ 174.33, 146.52, 108.60, 99.81, 85.34, 83.26, 79.23, 72.52, 51.60, 37.78, 34.16, 29.27, 25.99 (6C), 25.02, 24.68, 18.33, 11.70, 0.05 (3C), −4.34, −4.72; HRESIMS *m*/*z* 457.2564 [M + Na]^+^ (calcd for C_24_H_42_O_3_Si_2_Na, 457.2565).

#### Methyl (*S*,*E*)-7-((*tert*-butyldimethylsilyl)oxy)tetradeca-8-en-10,13-diynoate (**9**)

The TMS-protected acetylene **8** (110 mg, 0.253 mmol, 1.00 equiv) was dissolved in THF (3.83 mL) and EtOH (2.30 mL). A solution of AgNO_3_ (168 mg, 0.99 mmol, 3.90 equiv) in a mixture of EtOH and H_2_O (1:1, 2.70 mL) was added dropwise and stirred for 40 min. The reaction mixture changed from dark yellow to black after the addition of the AgNO_3_ solution. KCN (115.3 mg, 1.77 mmol, 7.00 equiv) was dissolved in H_2_O (1.95 mL) and added dropwise at room temperature (rt) (precipitation was observed during this stage). The reaction was stirred for 2 h, quenched by the addition of H_2_O (30 mL), and diluted by EtOAc (40 mL). The phases were separated, and the aqueous phase was extracted with EtOAc (3 × 30 mL). The combined organic phase was dried (Na_2_SO_4_), filtrated, and concentrated in vacuo. The crude product thus obtained was purified by flash chromatography (SiO_2_, 5% EtOAc in heptane) to obtain product **9** (78.1 mg, 0.215 mmol, 85% yield) as a yellow oil: *R*_f_ (5% EtOAc in heptane, visualized by UV and KMnO_4_ stain) = 0.24; [α]_D_^25^ −0.89 (*c* 0.1, CH_2_Cl_2_); ^1^H NMR (600 MHz, CDCl_3_) δ 6.08 (dd, *J* = 15.8, 5.5 Hz, 1H), 5.62–5.57 (m, 1H), 4.14–4.10 (m, 1H), 3.66 (s, 3H), 3.30 (t, *J* = 2.5 Hz, 2H), 2.29 (t, *J* = 7.5 Hz, 2H), 2.08 (t, *J* = 2.7 Hz, 1H), 1.65–1.57 (m, 2H), 1.51–1.42 (m, 2H), 1.36–1.25 (m, 4H), 0.88 (s, 9H), 0.03 (s, 3H), 0.02 (s, 3H); ^13^C NMR (151 MHz, CDCl_3_) δ 174.31, 146.75, 108.41, 82.85, 79.45, 72.49, 68.97, 51.58, 37.78, 34.16, 29.28, 25.99 (6C), 25.03, 24.66, 18.34, 10.39, −4.34, −4.72; HRESIMS *m*/*z* 385.2169 [M + Na]^+^ (calcd for C_21_H_34_O_3_SiNa, 385.2169).

#### Methyl (7*S*,8*E*,15*E*,17*S*,19*Z*)-7,17-bis((*tert*-butyldimethylsilyl)oxy)docosa-8,15,19-trien-10,13-diynoate (**10**)

Vinyl iodide **3** (20 mg, 0.06 mmol, 1.0 equiv) was dissolved in THF (0.5 mL), and the solution was cooled to 0 °C before Pd(PPh_3_)_2_Cl_2_ (2.0 mg, 2.8 μmol, 5.0 mol %), CuI (0.7 mg, 3.9 μmol, 11 mol %), and Et_3_N (11 mg, 15 μL, 0.11 mmol, 2.0 equiv) were added. Alkyne **9** (50 mg, 0.14 mmol, 2.5 equiv) was dissolved in THF (0.3 mL) and added dropwise. The reaction mixture was allowed to slowly warm up to rt and stirred in the dark for 16 h. After completion, the reaction mixture was filtered through a plug of silica gel (15% EtOAc in heptane) and concentrated in vacuo. The crude product thus obtained was purified by flash chromatography (SiO_2_, 5% EtOAc in heptane) to obtain product **10** (20 mg, 0.04 mmol, 62%) as a pale yellow oil: *R*_f_ (10% EtOAc in heptane, visualized by UV and KMnO_4_ stain) = 0.40; [α]_D_^20^ −0.10 (*c* 0.3, CH_2_Cl_2_); ^1^H NMR (400 MHz, CDCl_3_) δ 6.10 (td, *J* = 15.7, 5.4 Hz, 2H), 5.67–5.59 (m, 2H), 5.50–5.41 (m, 1H), 5.36–5.26 (m, 1H), 4.21–4.08 (m, 2H), 3.66 (s, 3H), 3.42 (s, 2H), 2.34–2.16 (m, 4H), 2.06–1.98 (m, 2H), 1.67–1.57 (m, 2H), 1.51–1.42 (m, 2H), 1.38–1.24 (m, 4H), 0.95 (t, *J* = 7.5 Hz, 3H), 0.89 (s, 9H), 0.89 (s, 9H), 0.05 (s, 3H), 0.03 (s, 3H), 0.03 (s, 3H), 0.02 (s, 3H); ^13^C NMR (101 MHz, CDCl_3_) δ 174.36, 146.55, 146.15, 134.07, 124.19, 108.63, 108.55, 83.60, 83.58, 79.18, 79.14, 72.59, 72.52, 51.61, 37.80, 36.00, 34.17, 29.29, 25.99 (6C), 25.04, 24.69, 20.89, 18.36, 18.34, 14.30, 11.27, −4.33, −4.45, −4.67, −4.72; HRESIMS *m*/*z* 623.3921 [M + Na]^+^ (calcd for C_35_H_60_O_4_Si_2_Na, 623.3922).

#### Methyl (7*S*,8*E*,10*Z*,13*Z*,15*E*,17*S*,19*Z*)-7,17-bis((*tert*-butyldimethylsilyl)oxy)docosa-8,10,13,15,19-pentaenoate (**11**)

Diyne **10** (10 mg, 17 μmol, 1.0 equiv) was dissolved in EtOAc (1.0 mL) under argon. Quinoline (15 μL, 0.13 mmol) and 5% Pd/BaSO_4_ (10 mg) were added, and the flask was evacuated and refilled with hydrogen gas twice. The reaction mixture was stirred for 40 min before it was filtrated through a short plug of silica gel (15% EtOAc in heptane) and concentrated in vacuo. The crude product thus obtained was purified by flash chromatography (SiO_2_, 5% EtOAc in heptane) to obtain product **11** (7.1 mg, 12 μmol, 71%) as a clear oil: *R*_f_ (5% EtOAc in heptane, visualized by UV and KMnO_4_ stain) = 0.29; [α]_D_^20^ +0.54 (*c* 0.2, CH_2_Cl_2_); ^1^H NMR (400 MHz, CDCl_3_) δ 6.49–6.38 (m, 2H), 5.99 (t, *J* = 10.9, 2H), 5.72–5.59 (m, 2H), 5.46–5.31 (m, 4H), 4.22–4.09 (m, 2H), 3.66 (s, 3H), 3.07–3.03 (m, 2H), 2.32–2.27 (m, 3H), 2.26–2.15 (m, 2H), 2.06–2.00 (m, 2H), 1.65–1.59 (m, 2H), 1.52–1.43 (m, 2H), 1.34–1.29 (m, 3H), 0.95 (t, *J* = 7.5 Hz, 3H), 0.91 (s, 9H), 0.90 (s, 9H), 0.06(s, 3H), 0.05 (s, 3H), 0.04 (s, 3H), 0.03 (s, 3H); ^13^C NMR (101 MHz, CDCl_3_) δ 174.39, 137.72, 137.24, 133.66, 129.13, 129.09, 128.70, 128.67, 124.81, 124.30, 124.25, 73.25, 73.21, 51.60, 38.34, 36.45, 34.21, 29.32, 26.56, 26.05 (6C), 25.09 (2C), 20.89, 18.43, 18.41, 14.35, −4.10, −4.25, −4.55, −4.59; HRESIMS *m*/*z* 627.4233 [M + Na]^+^ (calcd for C_35_H_64_O_4_Si_2_Na, 627.4235).

#### Methyl (7*S*,8*E*,10*Z*,13*Z*,15*E*,17*S*,19*Z*)-7,17-dihydroxydocosa-8,10,13,15,19-pentaenoate (**2**)

The bis-TBS-protected intermediate **11** (7 mg, 12 μmol, 1.0 equiv) was azeotroped with 2-MeTHF (2 × 1 mL) and then cooled to 0 °C neat before a solution of AcCl in dry MeOH (0.13 mL, 1.8 μmol, 15 mol %) was added (the solution was prepared just prior to use by adding freshly distilled AcCl (3.0 μL) to dry MeOH (2.0 mL) under argon). The reaction mixture was stirred for 4 h at 0 °C. The reaction mixture was diluted with CH_2_Cl_2_ (0.3 mL) prior to neutralization with a 10% aqueous solution of NaHCO_3_ (20 μL). The phases were separated, and the organic phase was washed with H_2_O (0.2 mL), dried (Na_2_SO_4_), filtrated, and concentrated in vacuo. The crude product thus obtained was purified by flash chromatography (SiO_2_, 40% EtOAc in heptane) to afford RvD5_n-3 DPA_ methyl ester **2** (3.0 mg, 8 μmol, 68%) as a clear oil. The chemical purity (97%) was determined by HPLC analysis (Eclipse XDB-C18, MeOH/H_2_O, 76:24, 1.0 mL/min): *t*_r_(minor) = 14.29 and 18.62 min, and *t*_r_(major) = 15.63 min. *R*_f_ (40% EtOAc in heptane, visualized by UV and KMnO_4_ stain) = 0.24; [α]_D_^20^ +0.15 (*c* 0.2, MeOH); UV–vis (MeOH) λ_max_ 242 nm (log ε = 4.64); ^1^H NMR (400 MHz, CD_3_OD) δ 6.56 (ddd, *J* = 15.0, 11.1, 3.9 Hz, 2H), 6.00 (td, *J* = 10.8, 4.6 Hz, 2H), 5.68 (ddd, *J* = 15.7, 9.8, 6.6 Hz, 2H), 5.49–5.35 (m, 4H), 4.16–4.07 (m, 2H), 3.65 (s, 3H), 3.09 (t, *J* = 7.6 Hz, 2H), 2.37–2.30 (m, 2H), 2.30–2.19 (m, 2H), 2.09–2.03 (m, 2H), 1.65–1.58 (m, 2H), 1.58–1.48 (m, 2H), 1.48–1.39 (m, 2H), 1.39–1.31 (m, 4H), 0.96 (t, *J* = 7.5 Hz, 3H); ^13^C NMR (151 MHz, CD_3_OD-higher-lock power) δ 176.00, 138.09, 137.54, 134.64, 130.26, 130.15, 129.64, 129.57, 126.25, 126.18, 125.53, 73.16, 73.15, 51.95, 38.22, 36.27, 34.75, 30.11, 27.38, 26.21, 25.98, 21.68, 14.51; HRESIMS *m*/*z* 399.2505 [M + Na]^+^ (calcd for C_23_H_36_O_4_Na, 399.2506).

### LC-MS/MS Matching Studies

Pooled human serum was purchased from Sigma (Poole UK). Mouse exudates were collected from the peritoneum of C57BL/6 mice and inoculated with 1 × 10^5^ CFU of *E. coli* (serotype O6:K2:H1; via intraperitoneal injection) 32 h after bacterial inoculation by injecting 4 mL of PBS into the peritoneum. Subsequently, two volumes of cold MeOH containing deuterium-labeled synthetic *d*_5_-Maresin 1 (250 pg) were added to human serum (0.5 mL) or the exudates (2 mL). Samples were stored at −20 °C for at least 45 min and then centrifuged at 2500 rpm for 10 min. Supernatant was collected and concentrated to ∼1.0 mL of MeOH content using a gentle stream of nitrogen gas (TurboVap LV system, Biotage). Solid phase extraction (SPE) was then performed through an ExtraHera automated extraction system (Biotage) adding 9 mL of aqueous pH 3.5 HCl solution. The acidified samples were then loaded onto conditioned C18 500 mg 200-0050-B cartridges (Biotage). Samples were washed with 4.0 mL of H_2_O and 5.0 mL of hexane, and products were eluted using 4.0 mL of methyl formate. Solvent was evaporated using a gentle stream of nitrogen (TurboVap LV, Biotage), and samples were resuspended in 40 μL of MeOH/H_2_O (1:1, vol/vol) solution. Samples were centrifuged at 2500 rpm for 5 min, and the supernatant was centrifuged again at 9900 rpm for 10 s, 4 °C.^19^

Following C18 SPE, samples were analyzed using a QTrap 6500+ (Sciex) MS system, coupled with a Shimadzu SIL-20AC HT autosampler and LC-20AD LC pumps. An Agilent C18 Poroshell column (150 × 4.6 × 2.7 μm) was used to separate lipid mediators. Using a constant flow rate of 0.5 mL/min, the eluent gradient started at 20:80:0.01 (vol/vol/vol) in MeOH/H_2_O/acetic acid for 0.2 min, which was ramped to 50:50:0.01 (vol/vol/vol) over 12 s, maintained for 2 min, ramped to 80:20:0.01 (vol/vol/vol) over 9 min and maintained for 3.5 min, then ramped to 98:2:0.01 (vol/vol/vol) and maintained for 5.5 min.

To obtain synthetic RvD5_n-3 DPA_ (**1**) for LC/MS-MS and receptor studies, synthetic **2** (10 μg) was incubated with 1 N LiOH (50 μL) in THF (0.5 mL) for 2 h at rt, and then H_2_O (15 μL) was added and THF evaporated using a gentle stream of nitrogen. The obtained material **1** was used as such. Reference material was obtained as detailed in ref (15). The identity of RvD5_n-3 DPA_ in the synthetic and biological matrices was determined using MRM with signature parent ion (Q1, *m*/*z* 361) and characteristic daughter ions (Q3, *m*/*z* 199, 143 or 263) coupled with an enhanced product ion (EPI) scan. For synthetic **1**, UV data were in accord with the literature.^7b,15^

### Biological Experiments

The experiments strictly adhered to UK Home Office regulations (Guidance on the Operation of Animals, Scientific Procedures Act, 1986) and Laboratory Animal Science Association (LASA) Guidelines (Guiding Principles on Good Practice for Animal Welfare and Ethical Review Bodies, 3rd Edition, 2015) and according to protocols detailed in a UK Home Office approved protocol (P998AB295).

### Impedance Assays

GPR101 receptor activation by synthetic RvD5_n-3 DPA_ (**1**) was assessed by monitoring impedance changes across CHO cell monolayers using an xCelligence RTCA DP system (ACEA Biosciences). GPR101-overexpressing CHO cells were plated 1 day prior to experiments at 80,000 cells per well in impedance measurement plates (E-Plate16; ACEA Biosciences). Cells were then washed with serum-free DMEM/F-12 medium, and 0.1, 1, or 10 nM of synthetic **1** or vehicle control (0.05% ethanol) in DMEM/F-12 medium was added to cells. Real-time changes in impedance were assessed over a 30 min interval.

### PBMC and Neutrophil Isolation from Whole Blood

Venous peripheral blood was collected from healthy volunteers after giving written consent in accordance with a Queen Mary Ethics of Research Committee-approved (QMERC22.331) study proposal. Human peripheral blood mononuclear cells (PBMCs) and neutrophils were isolated by Histopaque-1077 density centrifugation as follows: red blood cells (RBCs) were sedimented using 6% (w/v) dextran (MW 425,000–575,000; Sigma) followed by incubation at room temperature for 20 min. RBC-depleted upper layers were transferred to 50 mL tubes containing Histopaque-1077 (Sigma) and centrifuged for 30 min at 400*g* without a brake to separate PBMCs and neutrophil layers. Remaining RBCs in the neutrophil layer were lysed by incubating in 9 volumes of ice-cold ddH_2_O for 30 s followed by addition of 1 volume 10× Hank’s balanced salt solution (HBSS). Cells were enumerated using a hemacytometer with Turk’s stain and directly used in experiments (for neutrophils), or PBMCs were seeded in 10 cm plates at 30 million cells per plate and differentiated to macrophages by incubation in RPMI-1640 medium containing 10% human serum (Sigma) and 20 ng/mL GM-CSF (Peprotech) for 7 days.

### Phagocytosis Assays

For phagocytosis assays, pHrodo Green-conjugated *S. aureus*, pHrodo Red-conjugated *E. coli*, or pHrodo Red-conjugated zymosan bioparticles (Invitrogen) were opsonized by incubating with 0.3 mg/mL human gamma immunoglobins (Sigma) for 30 min at 37 °C in DPBS. Isolated neutrophils were stained in suspension with Hoechst 33342 (Invitrogen) for 15 min at 37 °C, followed by washing, resuspension in X-VIVO15 medium (Lonza) containing 2 mM l-glutamine (Sigma) and 1% penicillin/streptomycin solution (Sigma), and seeding in 96-well plates at 250,000 cells/well. Neutrophils were allowed to adhere for 30 min at 37 °C. For monocyte-derived macrophages, cells were seeded in 96-well plates at 50,000 cells/well in RPMI-1640 medium containing 2 mM l-glutamine and 1% penicillin/streptomycin solution, allowed to adhere overnight, and stained with Hoechst 33342 for 15 min at 37 °C. For all experiments, cells were then treated with 0.001, 0.01, 0.1, 1, or 10 nM of synthetic RvD5_n-3 DPA_ (**1**) or vehicle control (0.05% ethanol) in X-VIVO15 (for neutrophils) or RPMI-1640 (for macrophages) medium for 15 min at 37 °C. After incubation, opsonized pHrodo Green-conjugated *S. aureus* (5 μg/well), pHrodo Red-conjugated *E. coli* (5 μg/well), or pHrodo Red-conjugated zymosan (2.5 μg/well) was directly added to the wells. The increase in the pHrodo Red/Green signal over time, indicative of bioparticle phagocytosis, was quantified using a Celldiscoverer 7 high-content imaging system (Zeiss) over a 2 h period.

### Macrophage Phenotypic Marker Assessment by Flow Cytometry

For assessment of macrophage phenotypic markers, PBMCs were obtained as described above and seeded in low-adhesion 12-well plates (Greiner) at 800,000 cells per well. Cells were then differentiated to macrophages by incubation in RPMI-1640 medium containing 10% human serum and 20 ng/mL GM-CSF for 7 days in the presence of 10 nM synthetic RvD5_n-3 DPA_ (**1**) or a vehicle control (0.05% ethanol). After 7 days, the cells were lifted from the plates by washing with DPBS followed by incubation in DPBS containing 5 mM EDTA and gentle pipetting. Cells were then incubated in 8 mg/mL human gamma immunoglobulins to block aspecific antibody binding and stained with fluorescently conjugated antibodies against human macrophage phenotypic markers as follows: BV421-conjugated anti-MerTK, BV650-conjugated anti-CD80, BV711-conjugated anti-CD64, AF488-conjugated anti-CD68, PerCP-Cy5.5-conjugated anti-CD206, PE-CF594-conjugated anti-CD163, PE-Cy7-conjugated anti-CD32, APC-conjugated anti-CD142, and APC-Cy7-conjugated anti-CD14 (all Biolegend). Cells were then washed, fixed and permeabilized using the eBioscience Foxp3/transcription factor staining buffer set (Invitrogen), and stained with PE-conjugated anti-Arg1 (Invitrogen). Cells were then washed again, and staining was evaluated using a BD Fortessa II operated using BD FACSDiva. Results were analyzed using FlowJo v10.5 (TreeStar) and MetaboAnalyst 5.0 (https://www.metaboanalyst.ca/).

## Supporting Information Available

^1^H, ^13^C, COSY NMR, HRMS, and UV–vis data, HPLC chromatogram of RvD5_n-3 DPA_ methyl ester (**2**); HRMS as well as ^1^H and ^13^C NMR spectra of all synthetic intermediates (PDF)
Raw NMR data files (ZIP)
