# Supplementary material for: Stereoselective Synthesis, Pro-resolution, and Anti-inflammatory Actions of RvD5n-3 DPA
Source: J Nat Prod. 2023 Oct 25;86(11):2546–53. doi: 10.1021/acs.jnatprod.3c00769 (PMC10683074; doi:10.1021/acs.jnatprod.3c00769)
Supplement: Supplementary file 1 — np3c00769_si_001.pdf [file np3c00769_si_001.pdf]

Supporting information for Stereoselective Synthesis, Pro-resolution and Anti-inflammatory Bioactions of RvD5$_{n-3\ DPA}$

Karina Ervik,[a] Amalie F. Reinertsen,[a] Duco S. Koenis,[b] Jesmond Dalli[b] and Trond V. Hansen[a]

[a] *Department of Pharmacy, Section for Pharmaceutical Chemistry, University of Oslo, PO Box 1068, N-0316, Oslo, Norway.*

[b] *Lipid Mediator Unit, Center for Biochemical Pharmacology, William Harvey Research Institute, Barts and The London School of Medicine, Queen Mary University of London, Charterhouse Square, London EC1M 6BQ, UK*

Table of Contents

^{1}H and ^{13}C NMR-Spectra S-2

UV-Vis Spectrum S-8

HPLC Chromatograms S-10

HRMS Spectra S-13

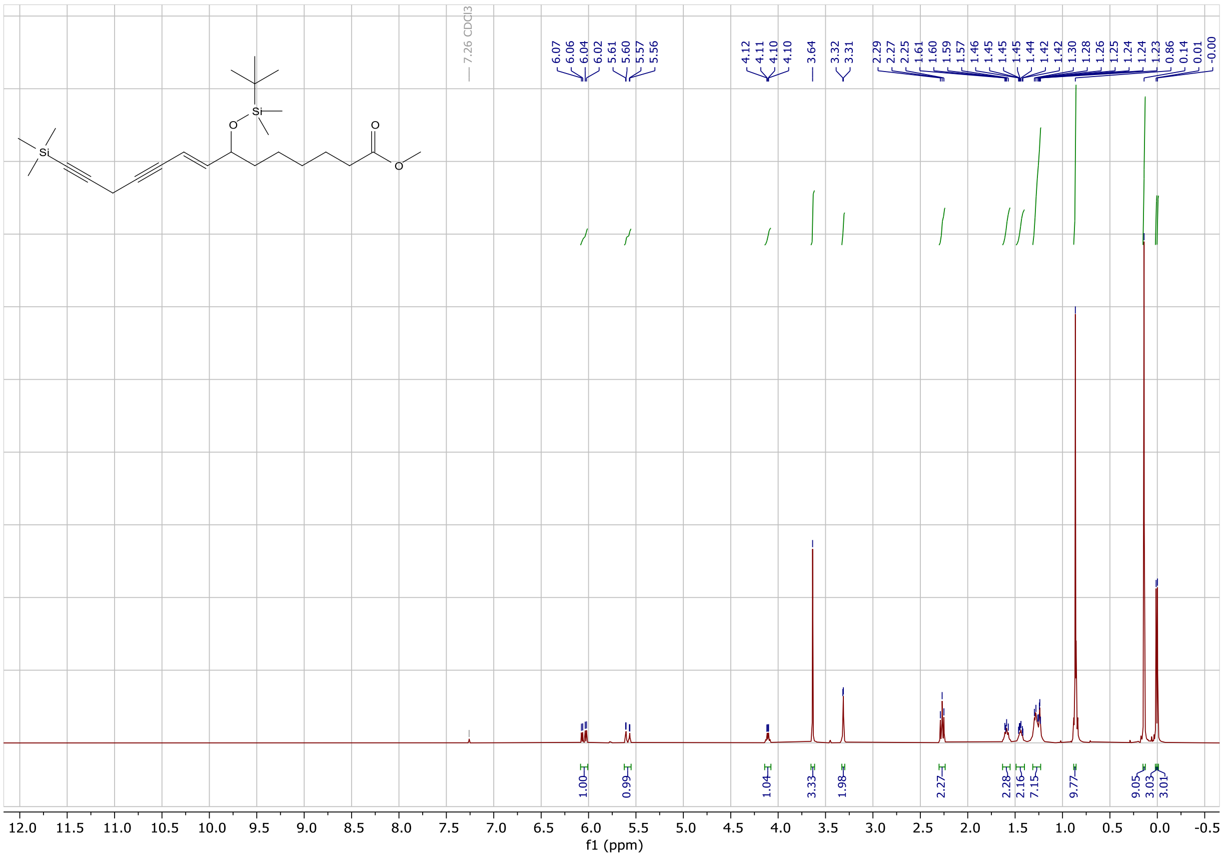

Figure S-1. ^{1}H NMR spectrum of compound **8**, $CDCl_3$, 400 MHz.

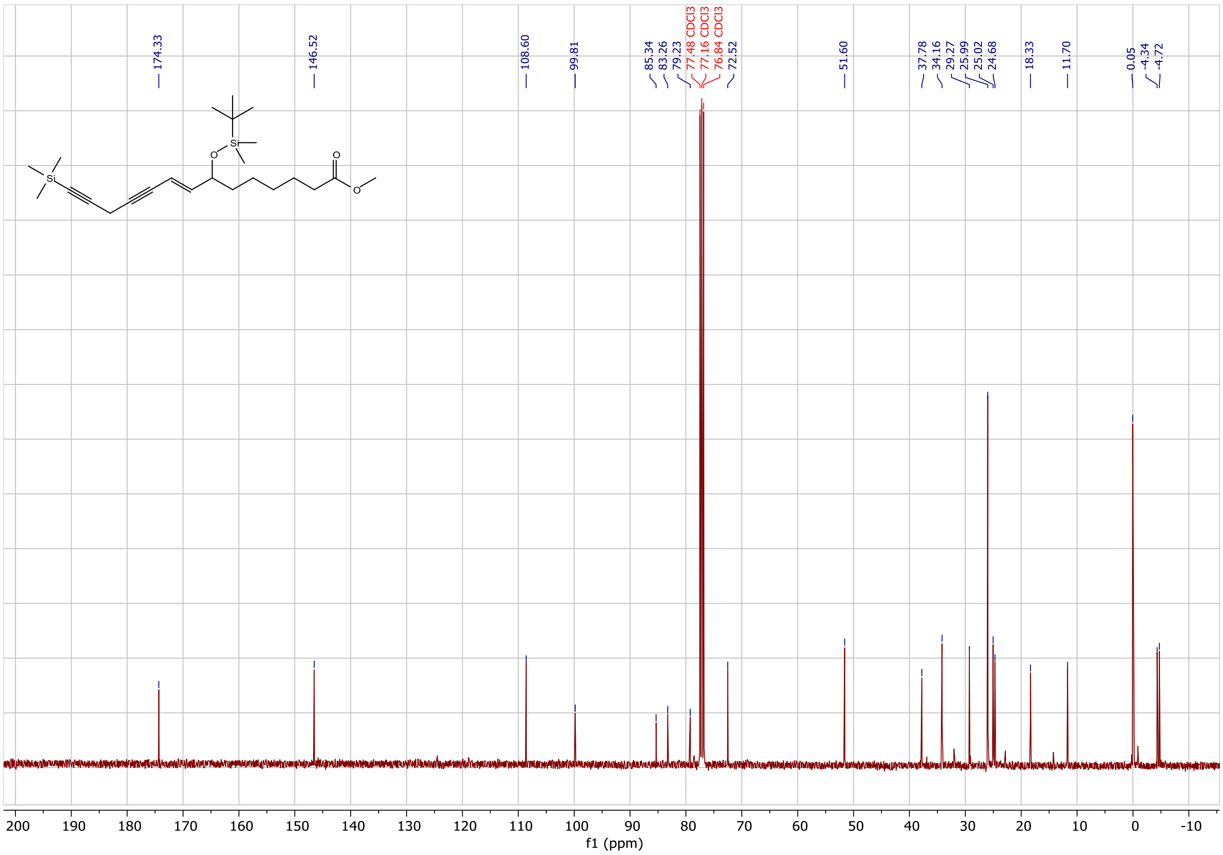

Figure S-2. ^{13}C NMR spectrum of compound **8**, $CDCl_3$, 100 MHz.

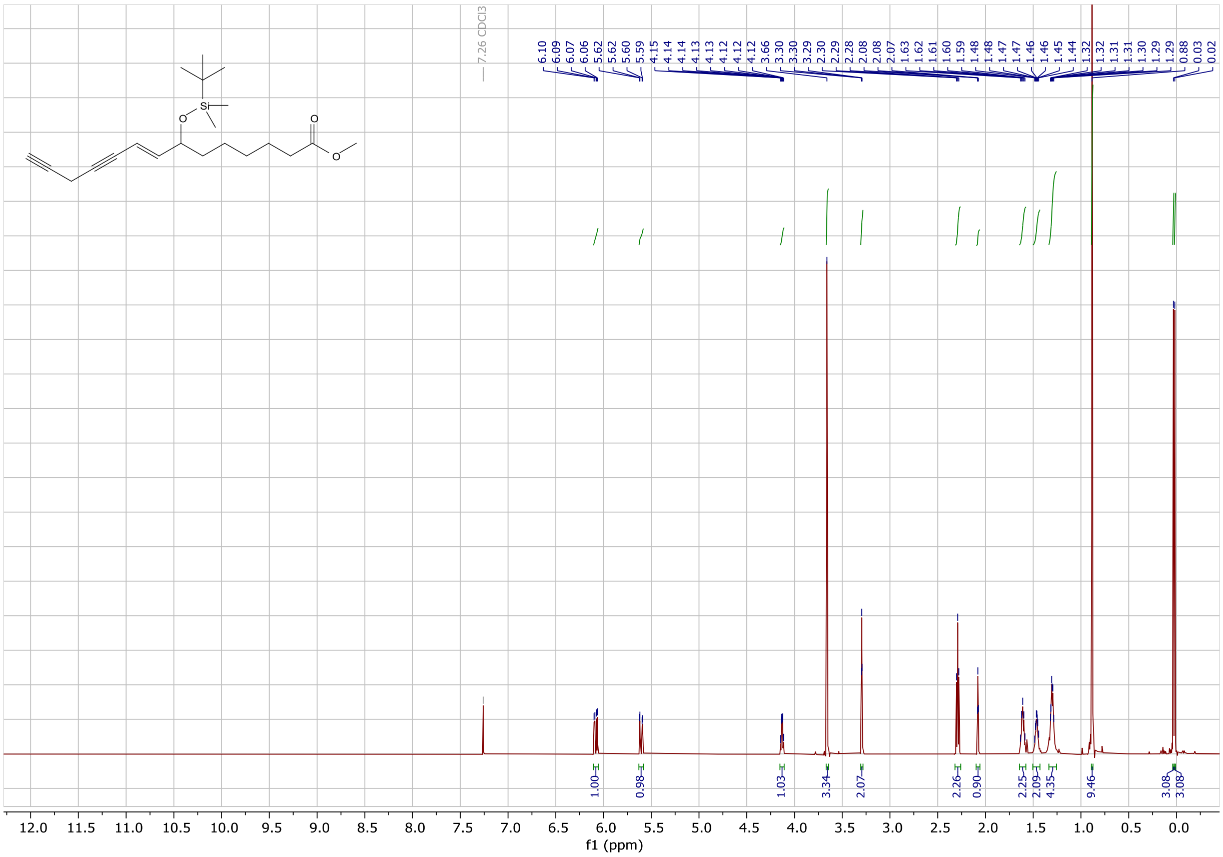

Figure S-3. ^{1}H NMR spectrum of compound **9**, $CDCl_3$, 400 MHz.

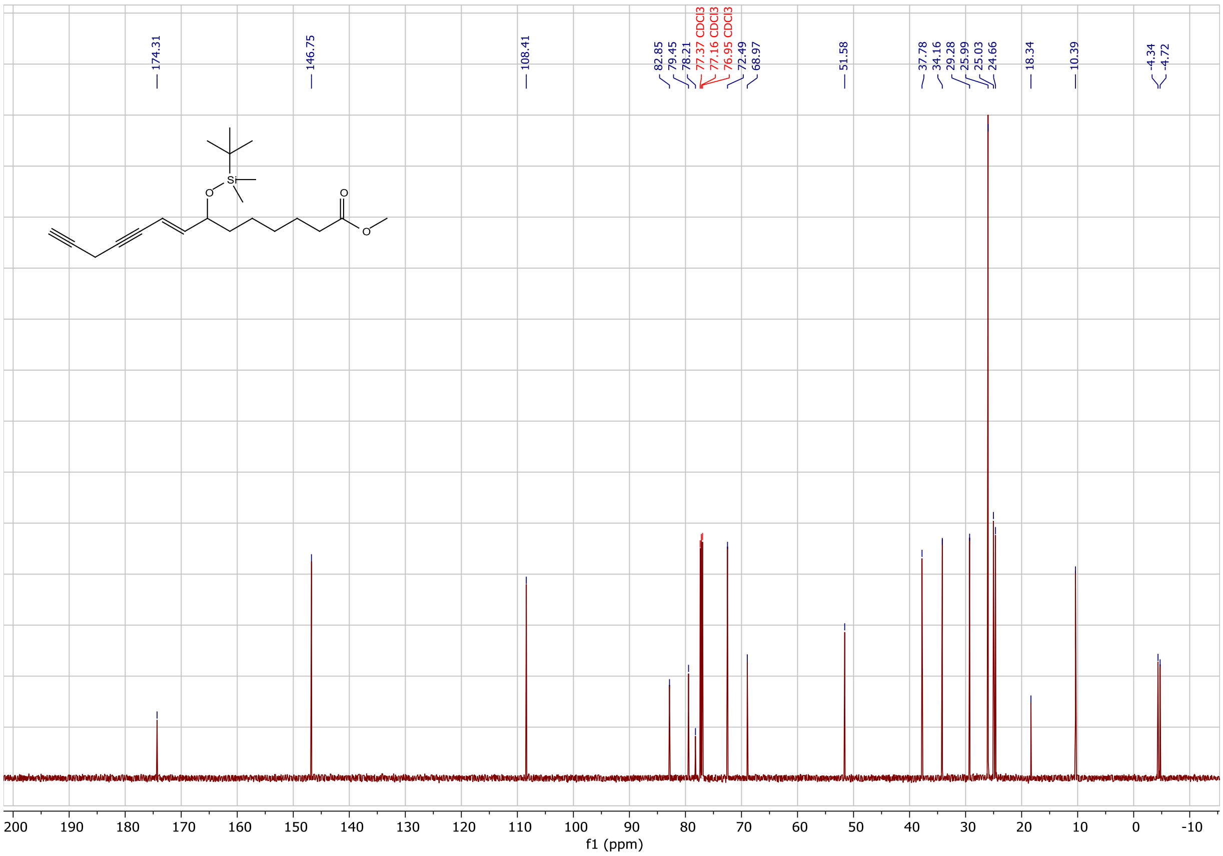

Figure S-4. ^{13}C NMR spectrum of compound **9**, $CDCl_3$, 100 MHz.

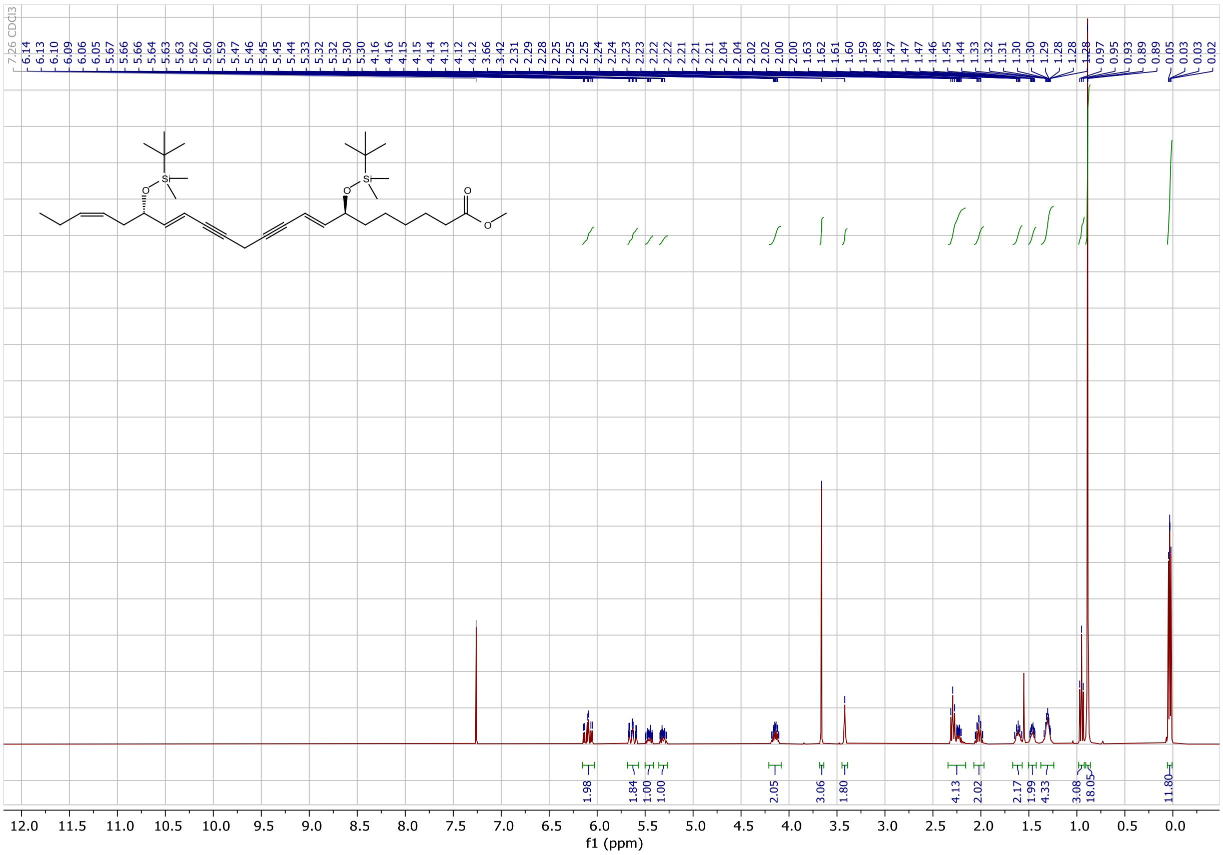

Figure S-5. ^{1}H NMR spectrum of compound **10**, $CDCl_3$, 400 MHz.

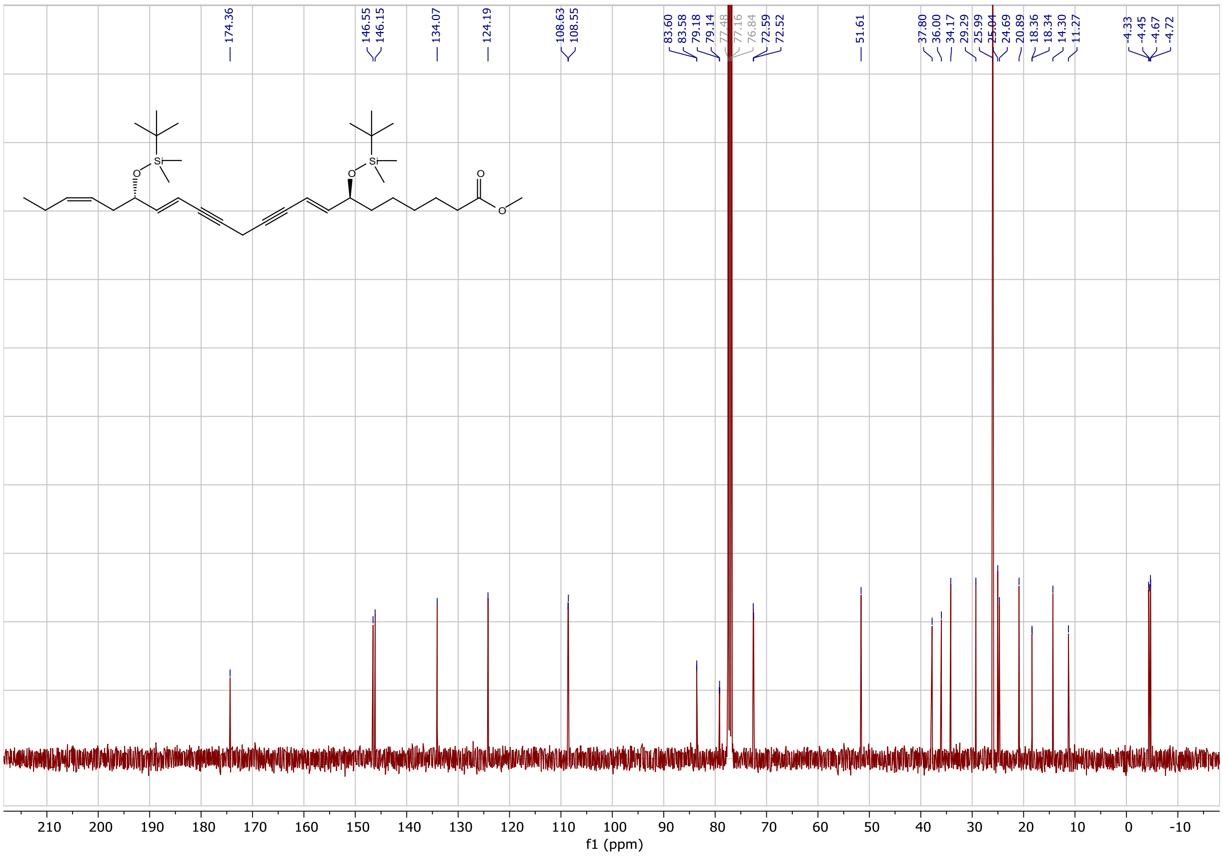

Figure S-6. ^{13}C NMR spectrum of compound **10**, $CDCl_3$, 100 MHz.

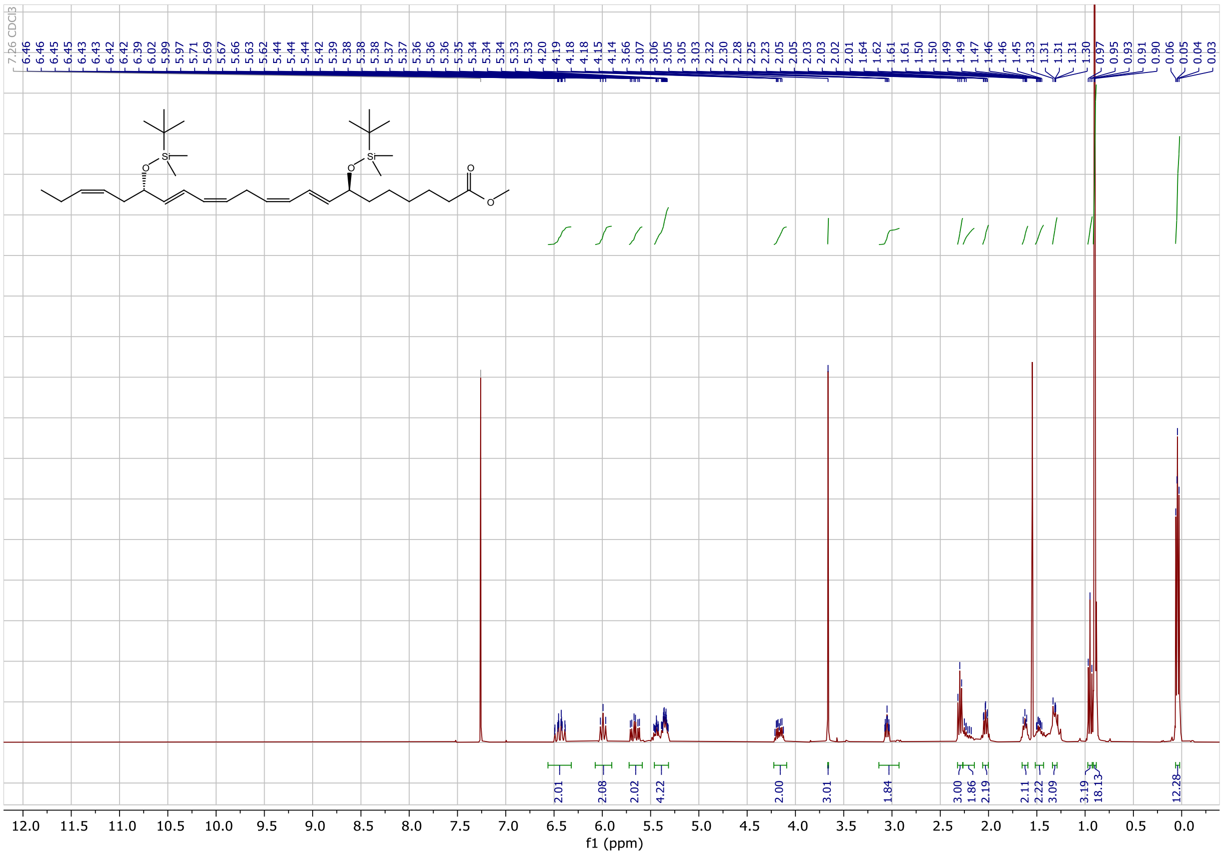

Figure S-7. ^{1}H NMR spectrum of compound **11**, $CDCl_3$, 400 MHz.

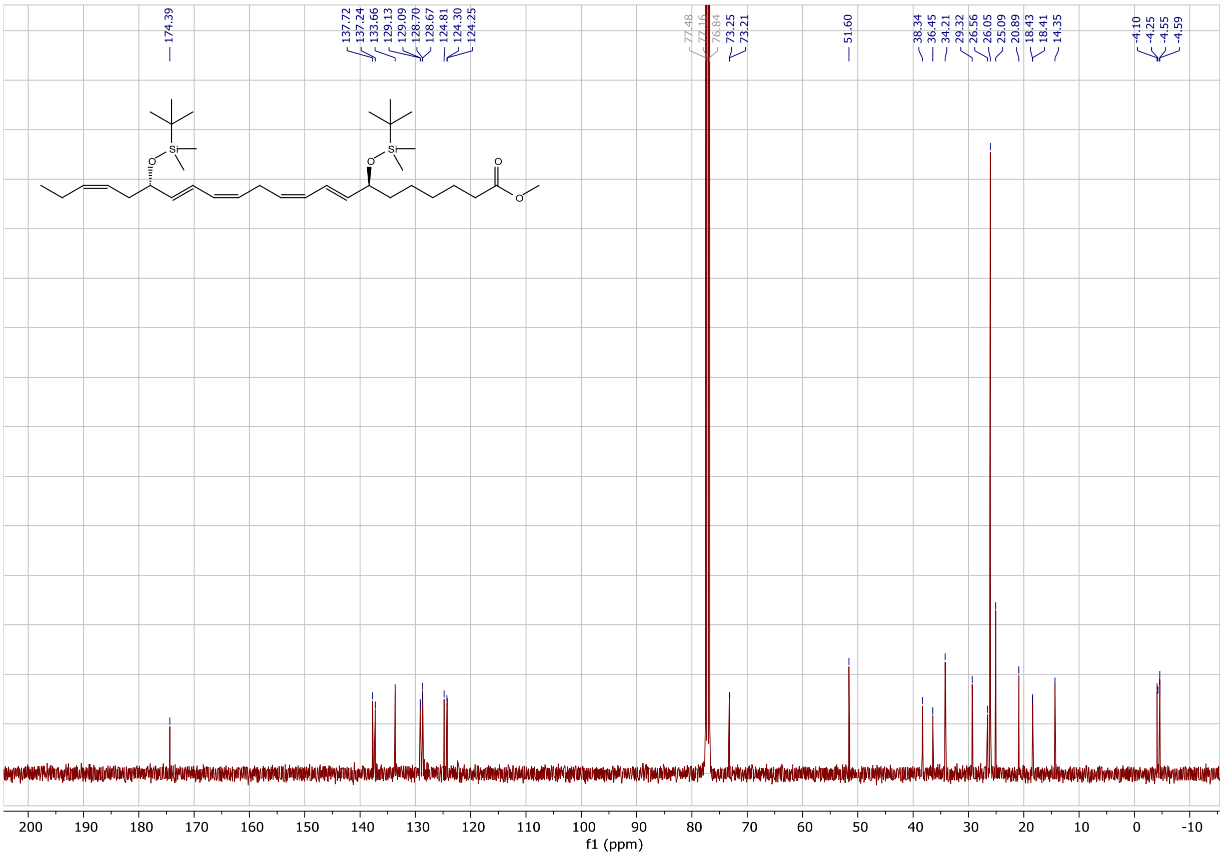

Figure S-8. ^{13}C NMR spectrum of compound **11**, $CDCl_3$, 100 MHz.

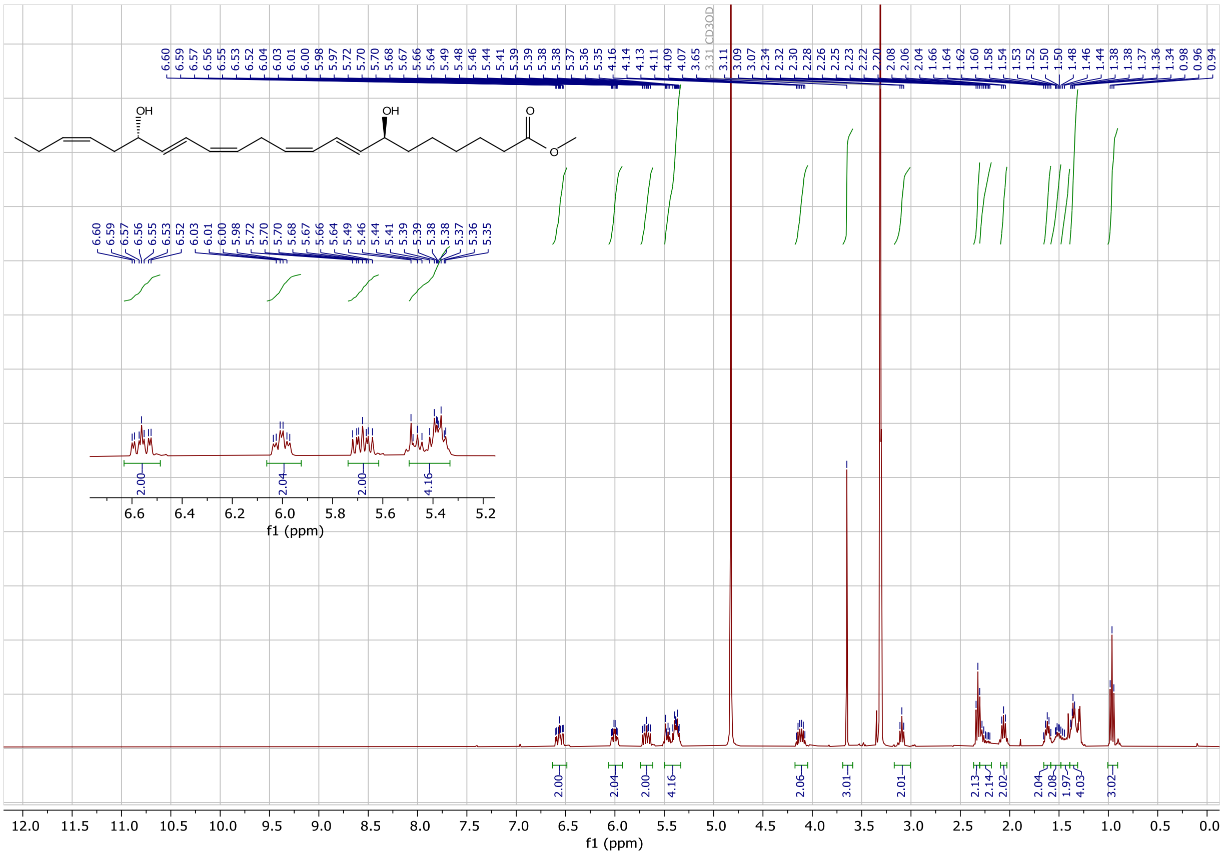

Figure S-9. ^{1}H NMR spectrum of compound RvD5$_{n\text{-}3\ DPA}$ methyl ester **2**, CD_3OD, 400 MHz.

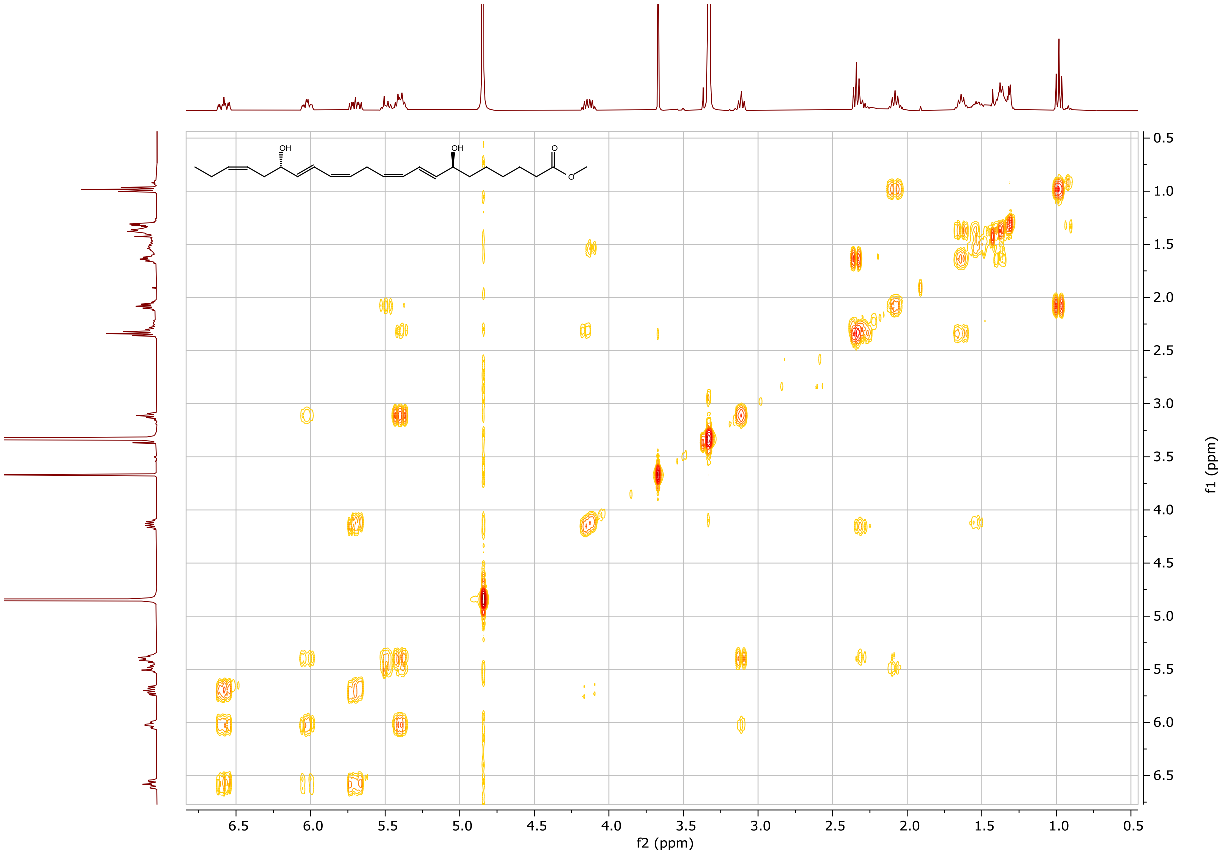

Figure S-10. COSY-spectrum of RvD5$_{n\text{-}3\ DPA}$ methyl ester **2**, CD_3OD, 400 MHz.

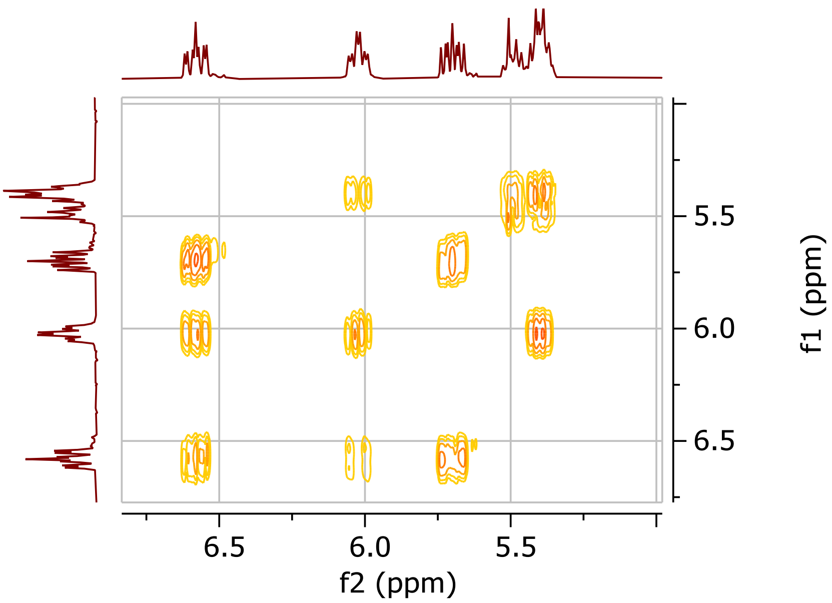

Figure S-11. Expansion of the olefinic area in the COSY NMR spectrum of RvD5$_{n\text{-}3\ DPA}$ methyl ester **2**.

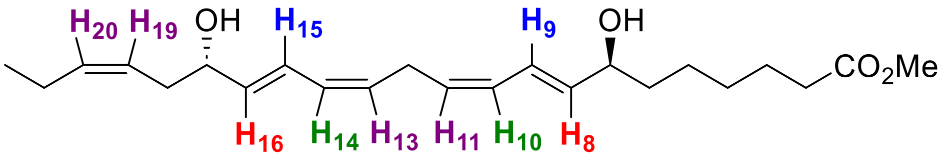

2

Position	*δ* (mult, *J*/Hz)
H_9, H_{15}	6.56 (ddd, *J* = 15.0, 11.1, 3.9 Hz, 2H)
H_{10}, H_{14}	6.00 (td, *J* = 10.8, 4.6 Hz, 2H)
H_8, H_{16}	5.68 (ddd, *J* = 15.7, 9.8, 6.6 Hz, 2H)
H_{11}, H_{13}, H_{19}, H_{20}	5.49 – 5.35 (m, 4H)

Table S-1. Interpretation of the double bond geometry in RvD5$_{n\text{-}3\ DPA}$ methyl ester (**2**).

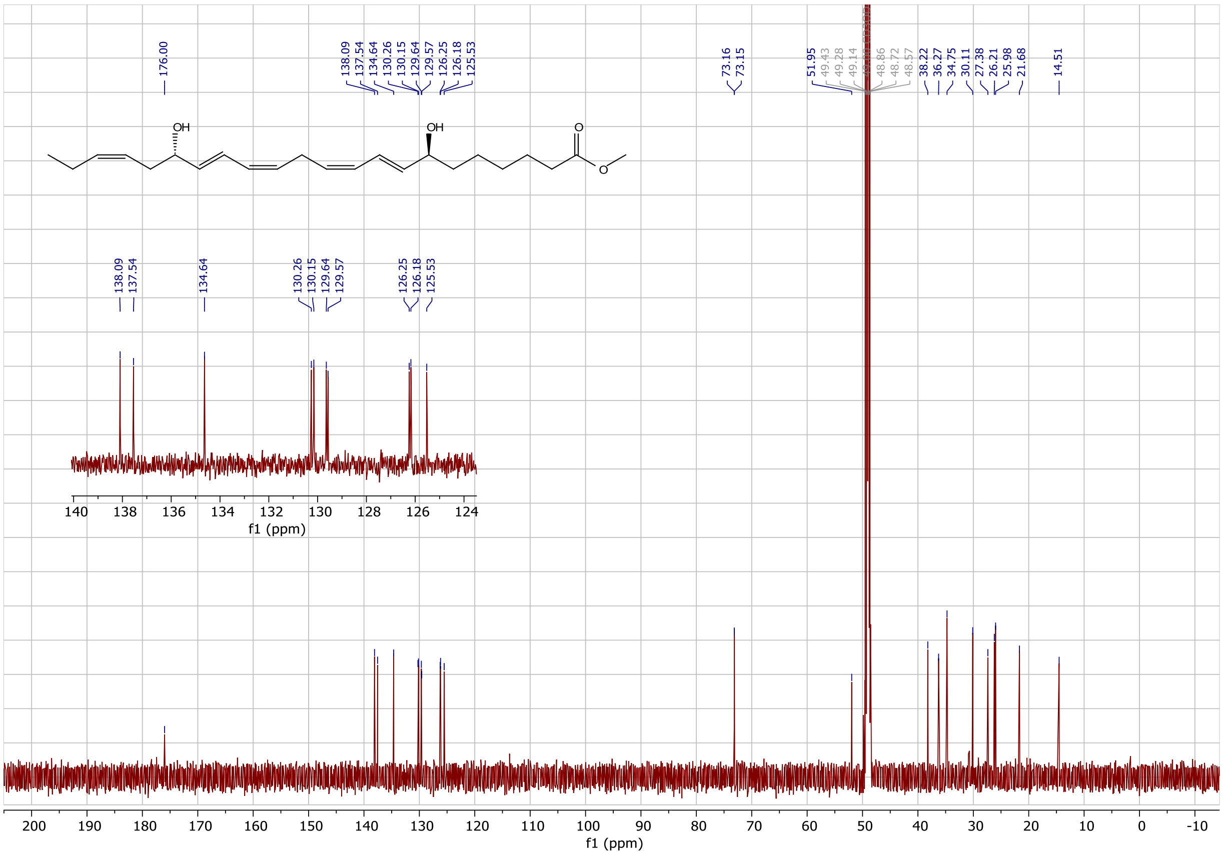

Figure S-12. ^{13}C NMR spectrum of RvD5$_{n\text{-}3\ DPA}$ methyl ester **2**, CD_3OD, 150 MHz.

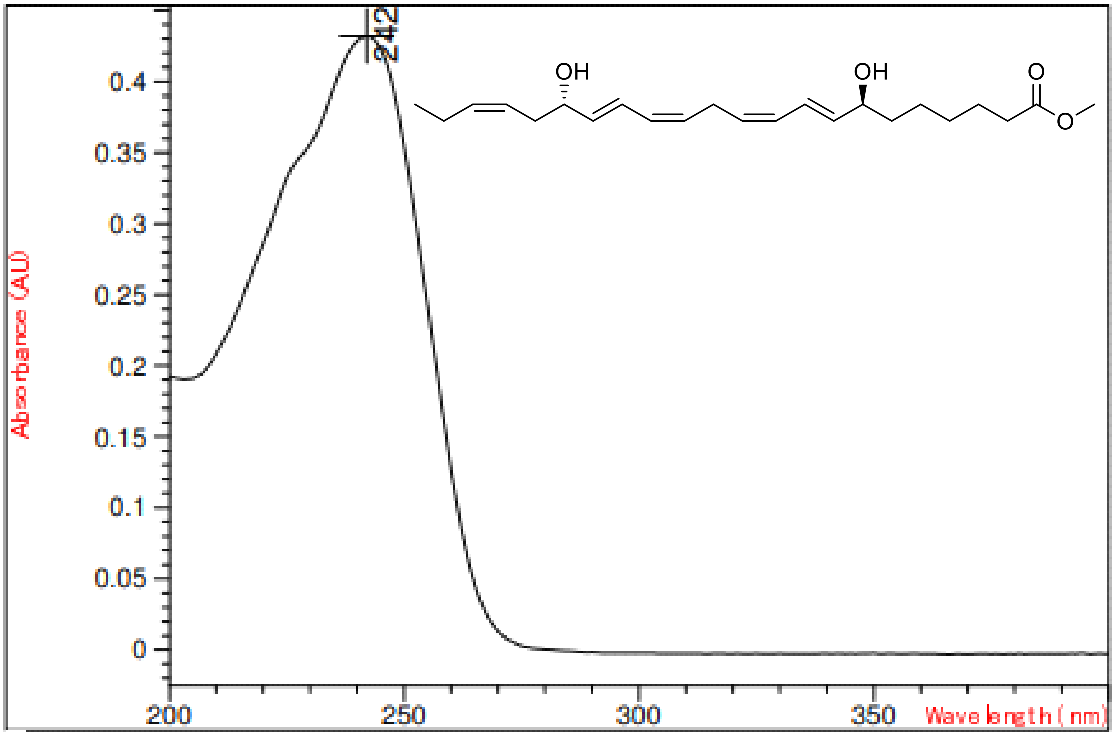

Figure S-13. UV-Vis of RvD5$_{n\text{-}3\ DPA}$ methyl ester **2** in MeOH.

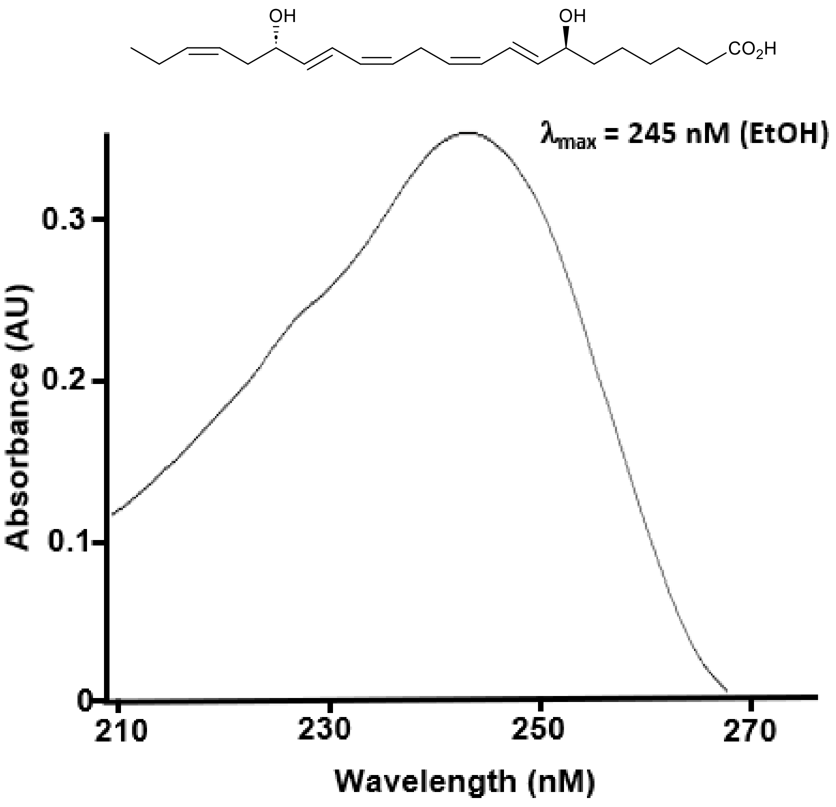

Figure S-14. UV-Vis of RvD5$_{n\text{-}3\ DPA}$ **1**.

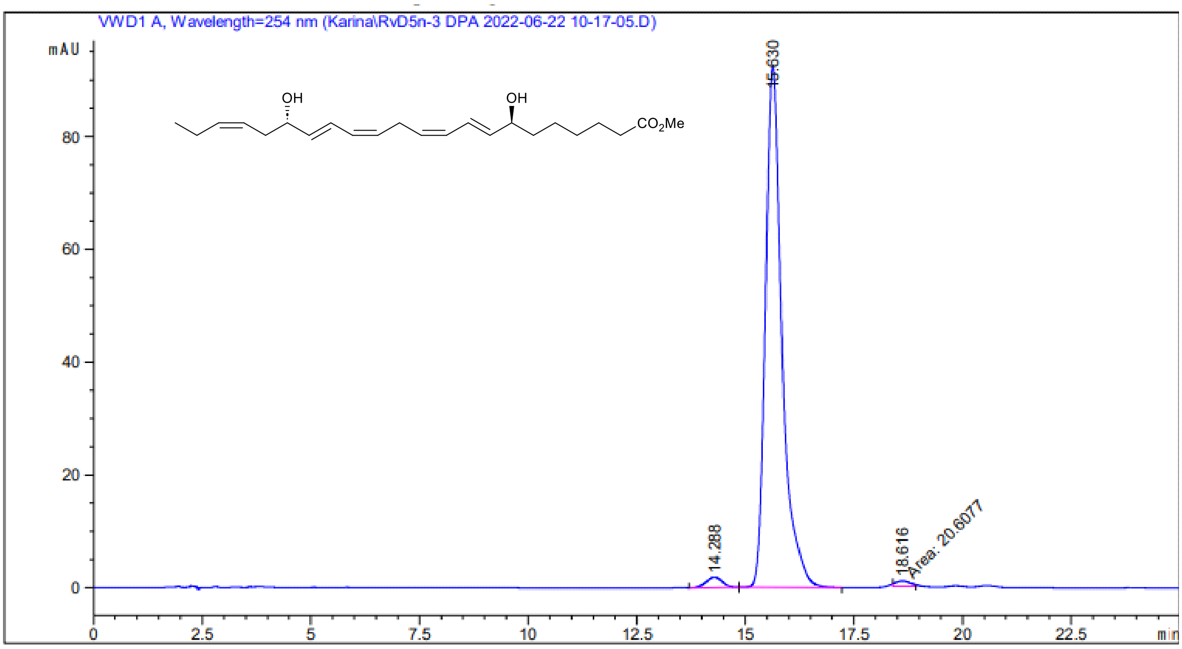


```
======================================================================
                        Area Percent Report
======================================================================

Sorted By              :      Signal
Multiplier             :      1.0000
Dilution               :      1.0000
Use Multiplier & Dilution Factor with ISTDs


Signal 1: VWD1 A, Wavelength=254 nm
```

Peak #	RetTime [min]	Type	Width [min]	Area [mAU*s]	Height [mAU]	Area %
1	14.288	BB	0.3929	48.07434	1.87654	1.9158
2	15.630	BB	0.3974	2440.66089	92.33601	97.2629
3	18.616	MM	0.3787	20.60766	9.06916e-1	0.8212

Figure S-15. HPLC chromatogram of RvD5$_{n\text{-}3\ DPA}$ methyl ester **2**.

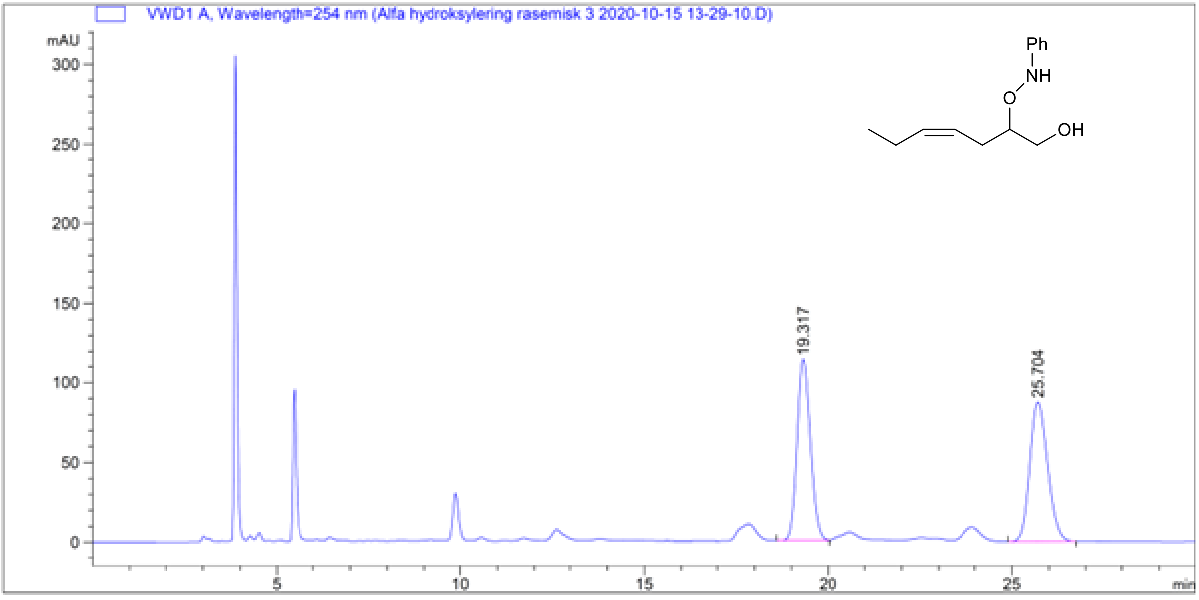


```
=====================================================================
                         Area Percent Report
=====================================================================

Sorted By             :      Signal
Multiplier            :      1.0000
Dilution              :      1.0000
Use Multiplier & Dilution Factor with ISTDs


Signal 1: VWD1 A, Wavelength=254 nm

Peak RetTime Type  Width     Area      Height     Area
  #   [min]        [min]   [mAU*s]     [mAU]       %
----|-------|----|-------|----------|----------|--------|
   1  19.317 BV    0.3891 2837.64722  113.32843  49.2803
   2  25.704 BB    0.5210 2920.53052   87.13389  50.7197

Totals :                  5758.17773  200.46232
```

Figure S-16. HPLC chromatogram of racemic α-aminoxylated alcohol intermediate of compound **7**.

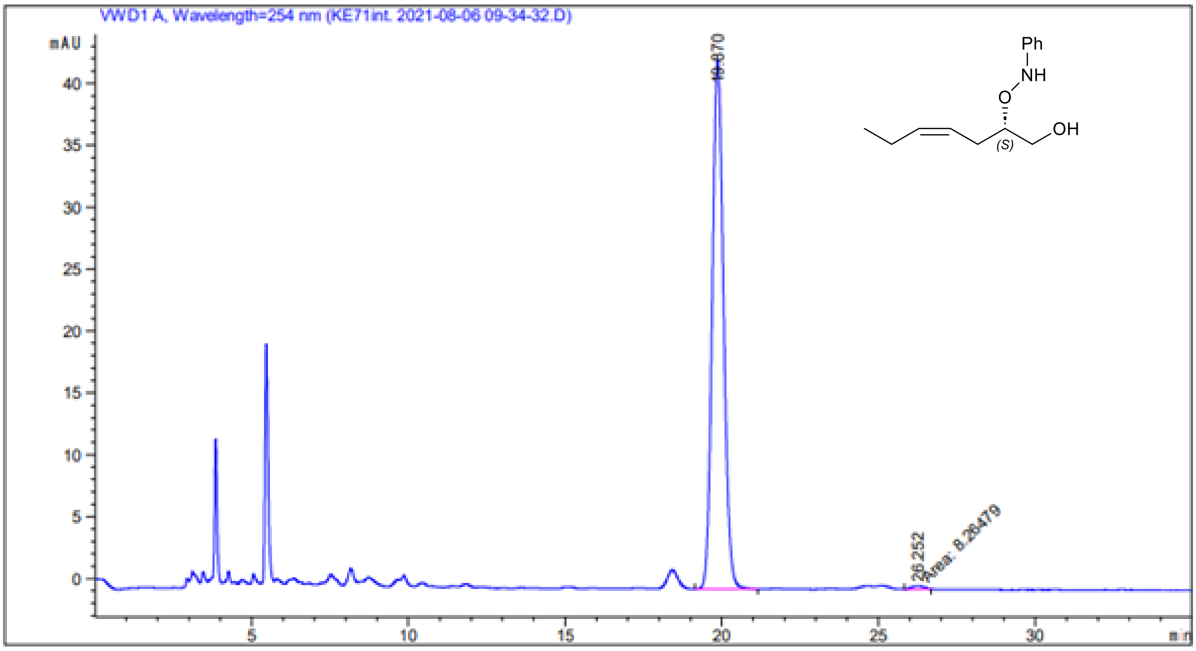


```
=====================================================================
                        Area Percent Report
=====================================================================

Sorted By             :      Signal
Multiplier            :      1.0000
Dilution              :      1.0000
Use Multiplier & Dilution Factor with ISTDs


Signal 1: VWD1 A, Wavelength=254 nm

Peak RetTime Type  Width     Area      Height     Area
  #   [min]        [min]   [mAU*s]     [mAU]        %
----|-------|----|-------|----------|----------|--------|
   1  19.870 BB    0.3840 1057.72974   42.69299 99.2247
   2  26.252 MM    0.4983    8.26479 2.76407e-1   0.7753
```

Figure S-17. HPLC chromatogram of optical α-aminoxylated alcohol intermediate of compound **7**.

Elemental Analysis Report

Analysis Info

Sample Name	KE79	Acquisition Date	9/27/2021 10:07:42 AM
Method	ESI_pos_50_1500_os.m	Analysis Name	D:\Data\maxis2021\18137.d

Acquisition Parameter

Source Type	ESI	Ion Polarity	Positive	Set Nebulizer	0.3 Bar
Focus	Not active	Set Capillary	3500 V	Set Dry Heater	200 °C
Scan Begin	50 m/z	Set End Plate Offset	-500 V	Set Dry Gas	4.0 l/min
Scan End	1500 m/z	Set Charging Voltage	2000 V	Set Divert Valve	Waste
		Set Corona	0 nA	Set APCI Heater	0 °C

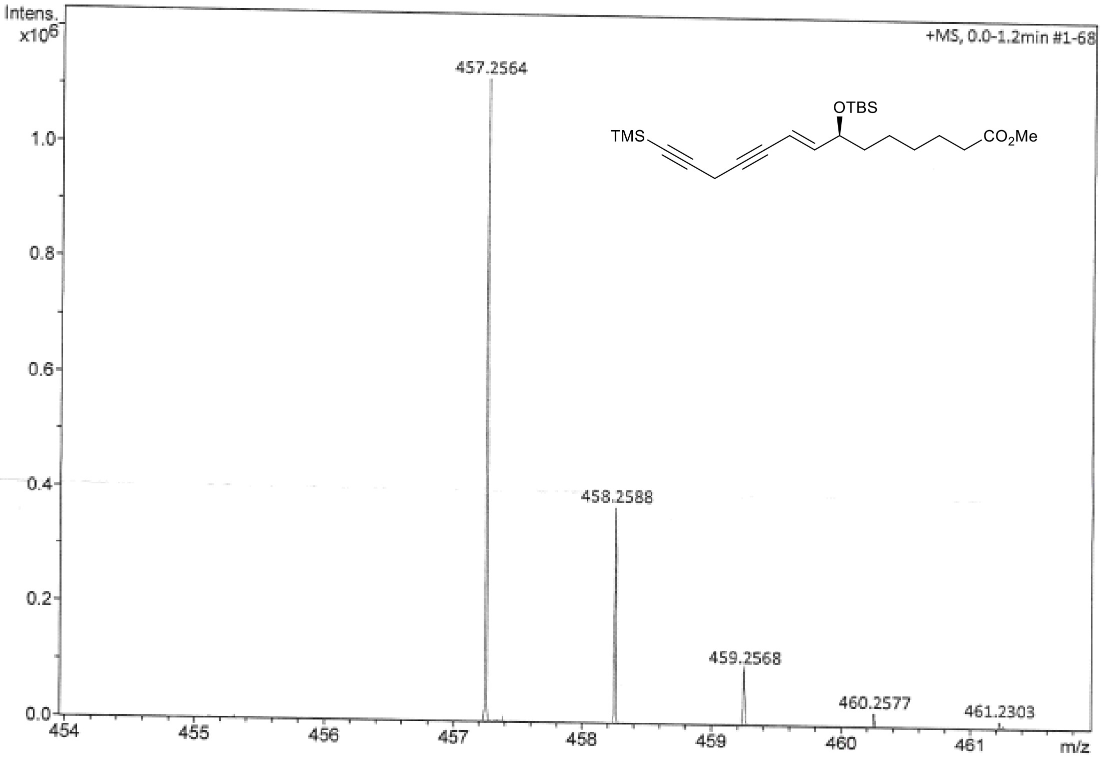

Meas. m/z	Ion Formula	m/z	err [ppm]
457.2564	C24H42NaO3Si2	457.2565	0.2
	C25H38NaO6	457.2561	-0.7
	C29H38NSi2	456.2537	-0.5
	C30H34NO3	456.2533	0.6
	C24H39NNaO4Si	456.2541	0.9

Figure S-18. HRMS spectrum of compound **8**.

Elemental Analysis Report

Analysis Info

Sample Name	KE80	Acquisition Date	05-Jan-22 3:48:15 PM
Method	ESI_pos_50_1500_os.m	Analysis Name	D:\Data\maxis2022\18362.d

Acquisition Parameter

Source Type	ESI	Set Capillary	3500 V	Set Nebulizer	0.3 Bar
Focus	Not active	Set End Plate Offset	-500 V	Set Dry Heater	200 °C
Scan Begin	50 m/z	Set Charging Voltage	2000 V	Set Dry Gas	4.0 l/min
Scan End	1500 m/z	Set Corona	0 nA	Set Divert Valve	Waste
				Set APCI Heater	0 °C

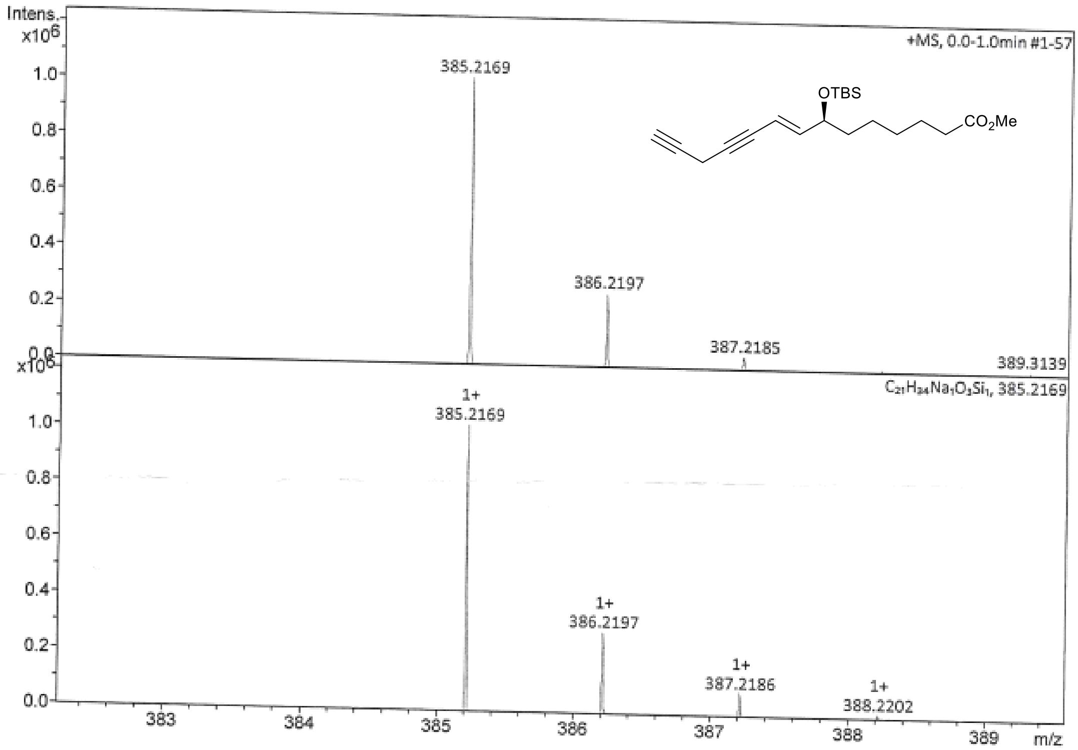

Meas. m/z	Ion Formula	m/z	err [ppm]
385.2169	C18H33N2O5Si	385.2153	-4.0
	C19H29N6OSi	385.2167	-0.6
	C27H29O2	385.2162	-1.7
	C21H34NaO3Si	385.2169	0.2

Figure S-19. HRMS spectrum of compound **9**.

Elemental Analysis Report

Analysis Info

Sample Name	KE81	Acquisition Date	07-Jan-22 9:54:56 AM
Method	ESI_pos_50_1500_os.m	Analysis Name	D:\Data\maxis2022\18371.d

Acquisition Parameter

Source Type	ESI	Set Capillary	3500 V	Set Nebulizer	0.3 Bar
Focus	Not active	Set End Plate Offset	-500 V	Set Dry Heater	200 °C
Scan Begin	50 m/z	Set Charging Voltage	2000 V	Set Dry Gas	4.0 l/min
Scan End	1500 m/z	Set Corona	0 nA	Set Divert Valve	Waste
				Set APCI Heater	0 °C

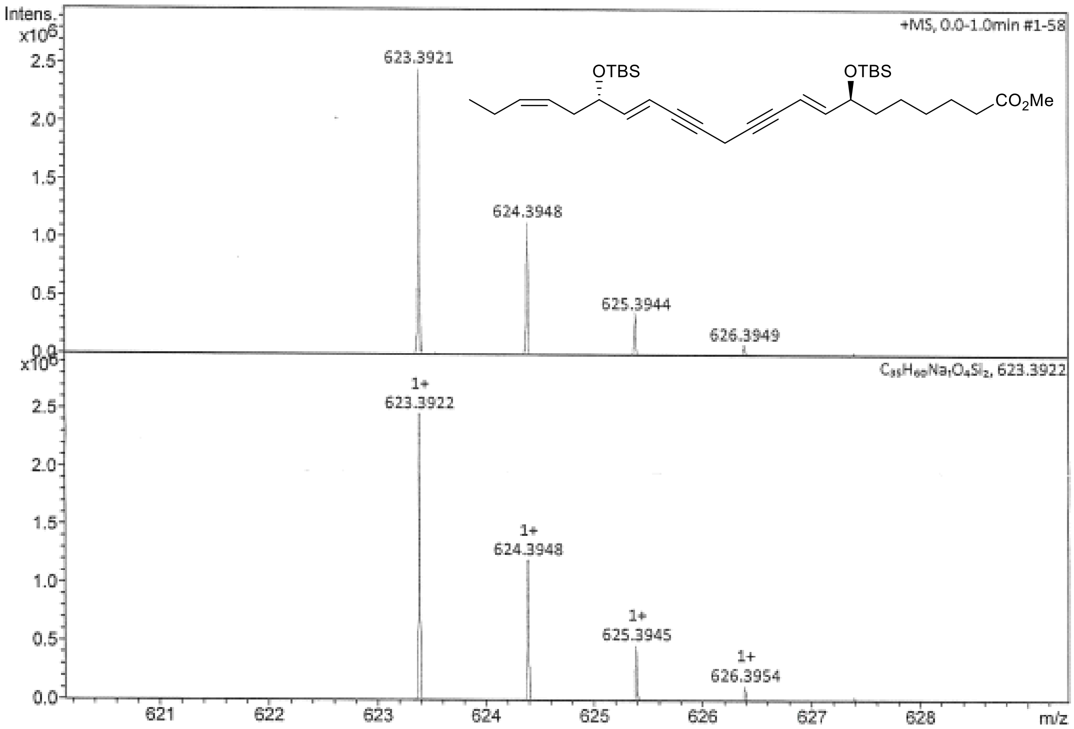

Meas. m/z	Ion Formula	m/z	err [ppm]
623.3921	C36H54N3O4Si	620.3878	0.8
	C41H55O3Si	623.3915	-1.0
	C34H51N6O5	623.3915	-1.0
	C40H56NOSi2	622.3895	-0.0
	C41H52NO4	622.3891	0.5
	C36H57N2O3Si2	621.3902	0.6
	C35H60NaO4Si2	623.3922	0.1
	C36H56NaO7	623.3918	-0.5
	C35H57NNaO5Si	622.3898	1.0
	C38H51N3NaO3	620.3823	-0.9

Figure S-20. HRMS spectrum of compound **10**.

Elemental Analysis Report

Analysis Info

Sample Name	KE107.1	Acquisition Date	10-Jun-22 1:33:06 PM
Method	ESI_pos_50_1500_os.m	Analysis Name	D:\Data\maxis2022\18912.d

Acquisition Parameter

Source Type	ESI	Set Capillary	3500 V	Set Nebulizer	0.5 Bar
Focus	Not active	Set End Plate Offset	-500 V	Set Dry Heater	200 °C
Scan Begin	50 m/z	Set Charging Voltage	2000 V	Set Dry Gas	4.0 l/min
Scan End	1500 m/z	Set Corona	0 nA	Set Divert Valve	Waste
				Set APCI Heater	0 °C

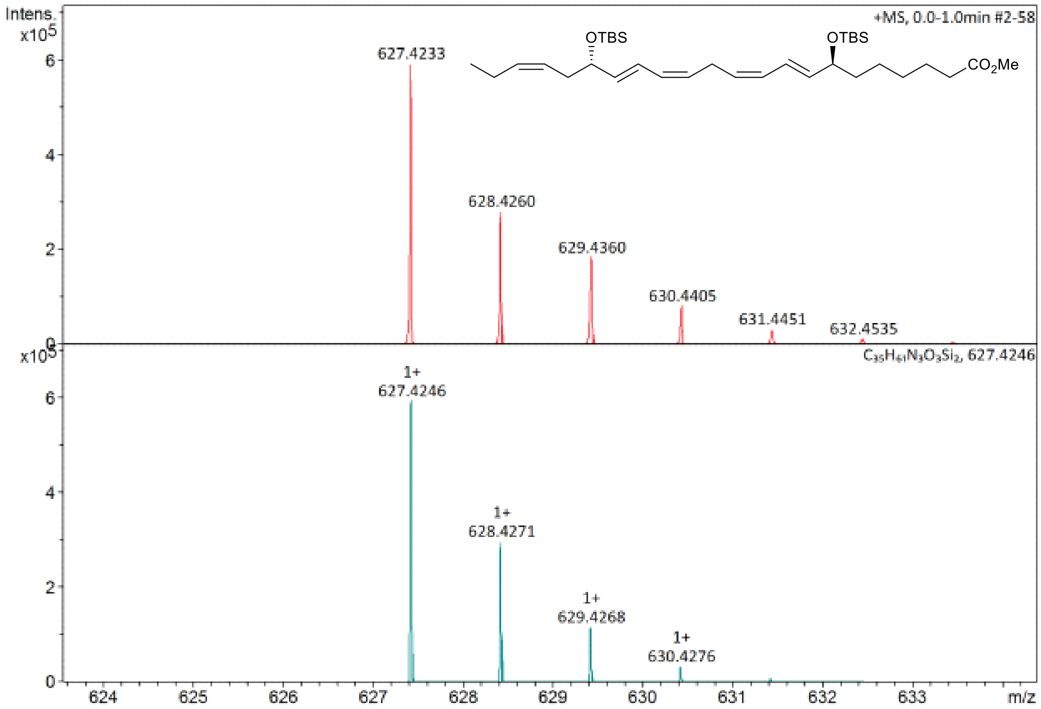

Meas. m/z	Ion Formula	m/z	err [ppm]
627.4233	C33H59N6O2Si2	627.4233	-0.1
	C35H64NaO4Si2	627.4235	0.3

Figure S-21. HRMS spectrum of compound **11**.

Elemental Analysis Report

Analysis Info

Sample Name	KE108	Acquisition Date	21-Jun-22 1:02:18 PM
Method	ESI_pos_50_1500_os.m	Analysis Name	D:\Data\maxis2022\18949.d

Acquisition Parameter

Source Type	ESI	Set Capillary	3500 V	Set Nebulizer	0.5 Bar
Focus	Not active	Set End Plate Offset	-500 V	Set Dry Heater	200 °C
Scan Begin	50 m/z	Set Charging Voltage	2000 V	Set Dry Gas	4.0 l/min
Scan End	1500 m/z	Set Corona	0 nA	Set Divert Valve	Waste
				Set APCI Heater	0 °C

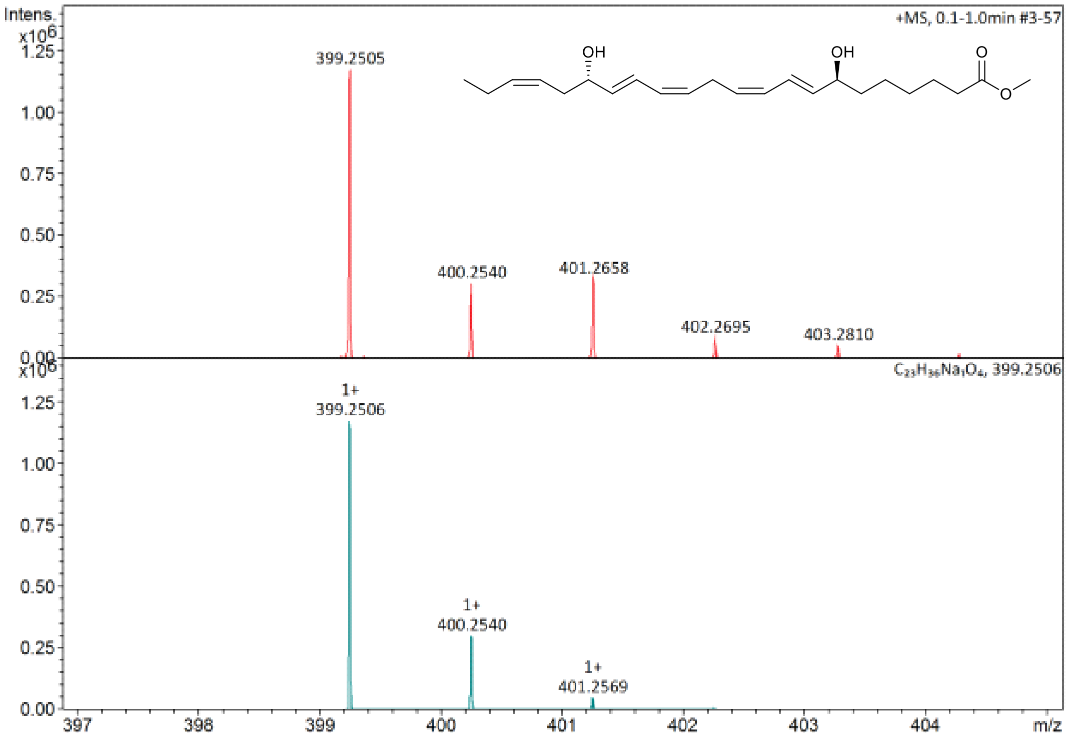

Meas. m/z	Ion Formula	m/z	err [ppm]
399.2505	C21H31N6O2	399.2503	-0.5
	C23H36NaO4	399.2506	0.2
401.2658	C21H33N6O2	401.2660	0.5
	C23H38NaO4	401.2662	1.2

Figure S-22. HRMS spectrum of RvD5$_{n\text{-}3\ DPA}$ methyl ester **2**.
