# Supplementary material for: Stereoselective Synthesis, Pro-resolution, and Anti-inflammatory Actions of RvD5n-3 DPA
Source: J Nat Prod. 2023 Oct 25;86(11):2546–53. doi: 10.1021/acs.jnatprod.3c00769 (PMC10683074; doi:10.1021/acs.jnatprod.3c00769)
Supplement: Supplementary file 2 — np3c00769_si_002.zip [file np3c00769_si_002.zip › NMR_FID/11/11-C/pdata/1/email_KE107_NN_11_1.pdf]

Pharmacy
C13CPD CDCl3 {D:\uio\AVneo400-05} karinaer 19

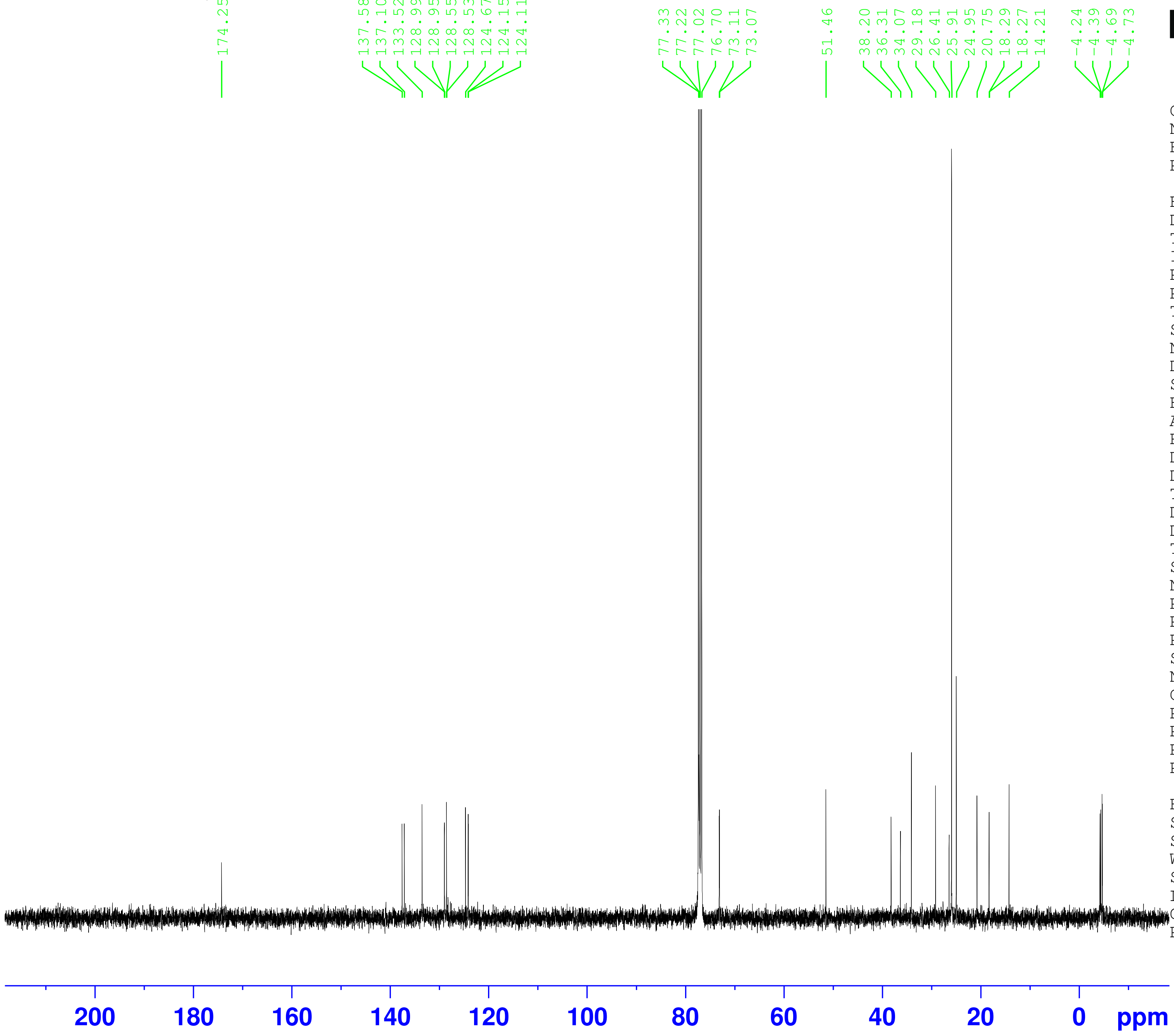

Current Data Parameters

NAME	KE107_NN
EXPNO	11
PROCNO	1

F2 - Acquisition Parameters

Date_	20220609
Time	0.53 h
INSTRUM	AVNeo400 Nanobay
PROBHD	Z163739_0427 (
PULPROG	zgpg30
TD	65536
SOLVENT	CDCl3
NS	3072
DS	4
SWH	23809.523 Hz
FIDRES	0.726609 Hz
AQ	1.3762560 sec
RG	71.8
DW	21.000 usec
DE	6.50 usec
TE	298.0 K
D1	2.00000000 sec
D11	0.03000000 sec
TD0	1
SFO1	100.6479773 MHz
NUC1	13C
P0	2.67 usec
P1	8.00 usec
PLW1	89.43399811 W
SFO2	400.2316009 MHz
NUC2	1H
CPDPRG[2	waltz65
PCPD2	90.00 usec
PLW2	21.45999908 W
PLW12	0.14783999 W
PLW13	0.07436100 W

F2 - Processing parameters

SI	32768
SF	100.6379135 MHz
WDW	EM
SSB	0
LB	1.00 Hz
GB	0
PC	1.40
