# Supplementary material for: Stereoselective Synthesis, Pro-resolution, and Anti-inflammatory Actions of RvD5n-3 DPA
Source: J Nat Prod. 2023 Oct 25;86(11):2546–53. doi: 10.1021/acs.jnatprod.3c00769 (PMC10683074; doi:10.1021/acs.jnatprod.3c00769)
Supplement: Supplementary file 2 — np3c00769_si_002.zip [file np3c00769_si_002.zip › NMR_FID/11/11-H/pdata/1/email_KE107_NN_10_1.pdf]

Pharmacy
PROTON CDCl3 {D:\uio\AVneo400-05} karinaer 19

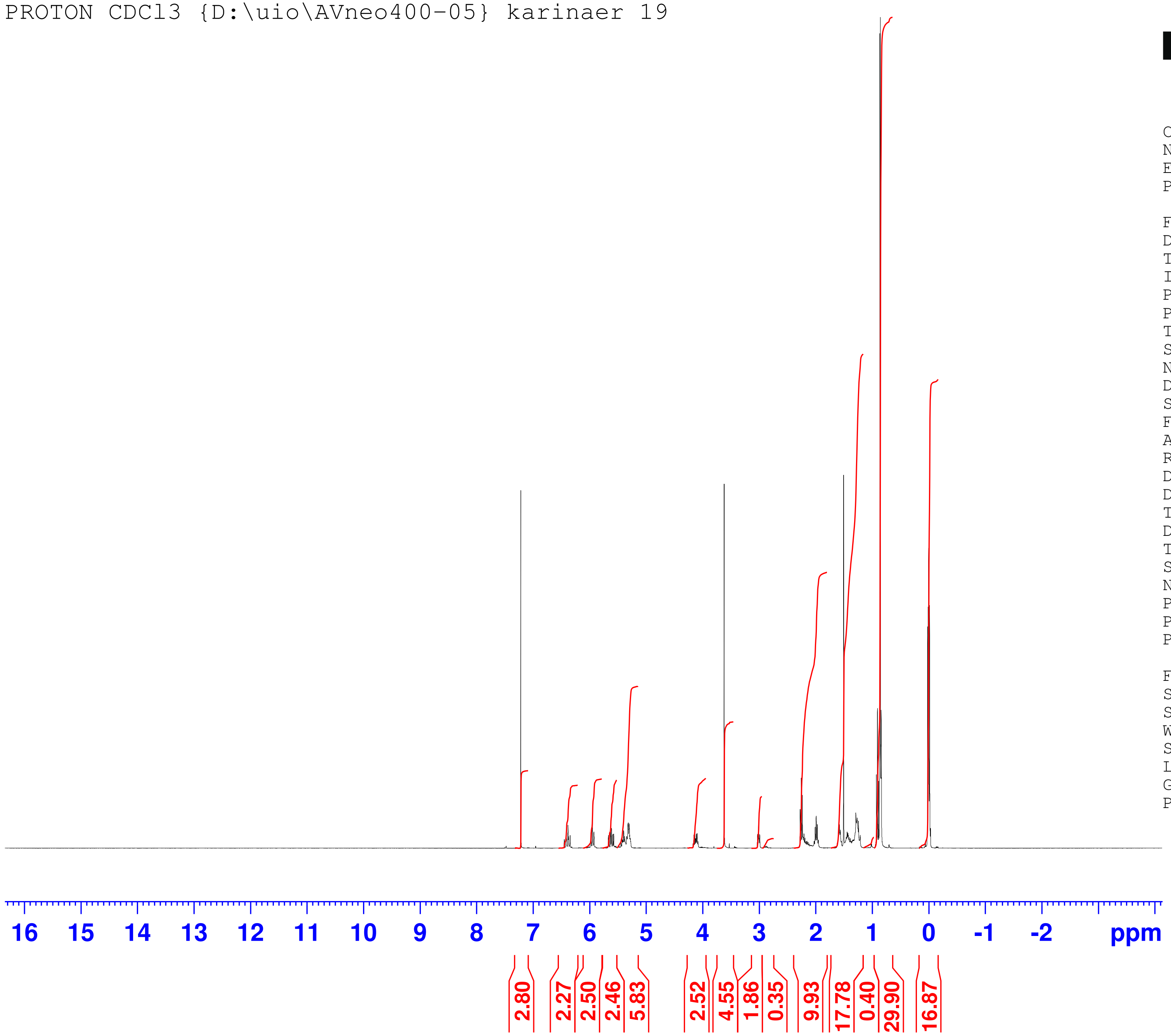

Current Data Parameters

NAME	KE107_NN
EXPNO	10
PROCNO	1

F2 - Acquisition Parameters

Date_	20220608
Time	21.56 h
INSTRUM	AVNeo400 Nanobay
PROBHD	Z163739_0427 (
PULPROG	zg30
TD	65536
SOLVENT	CDCl3
NS	48
DS	2
SWH	8196.722 Hz
FIDRES	0.250144 Hz
AQ	3.9976959 sec
RG	101
DW	61.000 usec
DE	13.98 usec
TE	298.0 K
D1	1.00000000 sec
TD0	1
SFO1	400.2324714 MHz
NUC1	1H
P0	2.49 usec
P1	7.47 usec
PLW1	21.45999908 W

F2 - Processing parameters

SI	65536
SF	400.2300275 MHz
WDW	EM
SSB	0
LB	0.30 Hz
GB	0
PC	1.00
