# Supplementary material for: Stereoselective Synthesis, Pro-resolution, and Anti-inflammatory Actions of RvD5n-3 DPA
Source: J Nat Prod. 2023 Oct 25;86(11):2546–53. doi: 10.1021/acs.jnatprod.3c00769 (PMC10683074; doi:10.1021/acs.jnatprod.3c00769)
Supplement: Supplementary file 2 — np3c00769_si_002.zip [file np3c00769_si_002.zip › NMR_FID/NMR-FID.docx]

NMR-FID

All NMR-spectra were recorded by a Bruker AVII 400, except 13C of compound **2**, which was recorded by Bruker AVII 600.

Acquisition software: TopSpin.

MestReNova was used to process and analyze the data.

Compounds:

**8**: 400 MHz 1H, 100 MHz 13C

**9**: 400 MHz 1H, 100 MHz 13C

**10**: 400 MHz 1H, 100 MHz 13C

**11**: 400 MHz 1H, 100 MHz 13C

**2**: 400 MHz 1H and COSY, 150 MHz 13C
